# Flexible single-layer ionic organic–inorganic frameworks towards precise nano-size separation

**DOI:** 10.1038/ncomms10742

**Published:** 2016-02-29

**Authors:** Liang Yue, Shan Wang, Ding Zhou, Hao Zhang, Bao Li, Lixin Wu

**Affiliations:** 1 State Key Laboratory of Supramolecular Structure and Materials, College of Chemistry, Jilin University, Changchun 130012, PR China; 2 Key Laboratory of Natural Resources of Changbai Mountain and Functional Molecules, Yanbian University, Yanji 133002, PR China

## Abstract

Consecutive two-dimensional frameworks comprised of molecular or cluster building blocks in large area represent ideal candidates for membranes sieving molecules and nano-objects, but challenges still remain in methodology and practical preparation. Here we exploit a new strategy to build soft single-layer ionic organic–inorganic frameworks via electrostatic interaction without preferential binding direction in water. Upon consideration of steric effect and additional interaction, polyanionic clusters as connection nodes and cationic pseudorotaxanes acting as bridging monomers connect with each other to form a single-layer ionic self-assembled framework with 1.4 nm layer thickness. Such soft supramolecular polymer frameworks possess uniform and adjustable ortho-tetragonal nanoporous structure in pore size of 3.4–4.1 nm and exhibit greatly convenient solution processability. The stable membranes maintaining uniform porous structure demonstrate precisely size-selective separation of semiconductor quantum dots within 0.1 nm of accuracy and may hold promise for practical applications in selective transport, molecular separation and dialysis systems.

## Abstract (web summary)

Membranes composed of ionic frameworks offer applications in nano-size separation thanks to their highly regular pores. Here, the authors devise such a system composed of polyoxometalates and organic pseudorotaxanes, and demonstrate their ability to separate mixed sized CdTe quantum dots.

## Graphical abstract

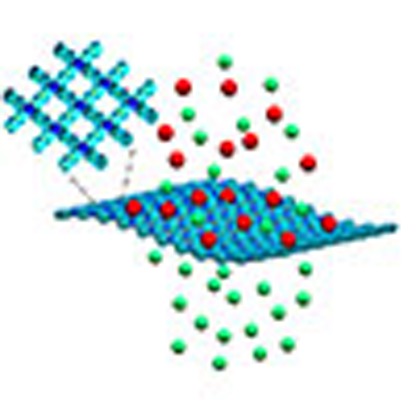

Ultrathin porous membranes^1^ have received increasing attention over recent years because of their superiorities, for example, permeation^2^,^3^, selective transport^4^ and molecular and nano-object sieving^5^ in the fields of chemical engineering^6^, biomedicine^7^, environment and energy^8^ and materials science^9^. Single-layer two-dimensional (2D) polymers^10^,^11^,^12^,^13^ with identical porosity and good processability represent a type of ideal porous membranes, but the big challenges still remain in structural design and synthetic methodology. Currently, in order to integrate discrete subunits into 2D connection, building block monomers must be specially designed with rigid planar structure^10^ or capable of preorganization into 2D mesostructure assisted by crystallization^11^,^12^,^13^, solid surface^14^,^15^ or interface^16^ support. These methods often bring about inevitable limitations, such as monomer shape and crosslinking reaction, structural damage in single-layer exfoliation and transferability. Therefore, a facile and general method to construct flexible single-layer 2D polymer in solution is still highly desired. As an applicable choice, supramolecular self-assembly^17^,^18^,^19^,^20^,^21^,^22^ based on intermolecular interactions has been proved to be a promising approach to integrate bridging monomers with connection nodes for 2D supramolecular polymer. Through coordination interaction, Kim's group prepared 2D polyrotaxane network with large cavities and channels, demonstrating a viable approach for modular porous solids^23^,^24^. Furthermore, Li and his coworkers developed single-layer 2D honeycomb supramolecular organic framework connected by inclusion interaction^25^. However, owing to the inevitably strong interlayer and weak in-layer interactions, a new synthetic methodology for single-layer soft frameworks in all desired area via a facile and rapid procedure is highly desired.

Polyoxometalates^26^,^27^,^28^,^29^ (POMs) are a kind of discrete metal-oxide clusters with precise chemical composition and structure. The rich architectures, uniform morphologies and multiple negative charges make POMs outstanding candidates for self-assembly^30^,^31^,^32^,^33^,^34^,^35^,^36^,^37^,^38^,^39^,^40^. When the POM cluster is considered as connection node and the electrostatic interaction between POM and cationic linker is applied as binding force, the construction of ionic organic–inorganic frameworks (IOIFs) in supramolecular network can be expected in principle. Based on the rational design of building block and ionic connection, the regular porosity can be predicted in supramolecular polymers. In addition, the non-direction preference of ionic bonding can bring flexibility for the self-assembled framework. Thus, the integration of identical porosity and convenient processability can be predicted to exist in the IOIFs. The tough barrier from the non-saturation and non-preferential direction of ionic bond needs to be overcome. Herein, we report a new strategy to construct free-standing single-layer 2D supramolecular polymer frameworks by means of synergistic ionic self-assembly of cationic α-cyclodextrin (CD)-based pseudorotaxane bridging sticks with the anionic PW_11_VO_40_^4−^ (PWV^4−^) cluster nodes in water. Because negative charges of the POM are delocalized and moveable^41^,^42^,^43^, cationic groups can spontaneously adjust their locations around the POM depending on steric effect and additional interaction. Therefore, the introduced CD provides steric guidance and lateral hydrogen bonding for the 2D arrangement of cationic sticks around polyanionic node. The cluster with four negative charges acts as both the crosslinker to bind with cations and the capper to lock pseudorotaxane sticks. Given the large-scale single-layer nanoporous sheets and convenient solution processability, ultrafiltration membranes (pore size 3.4–4.1 nm) are prepared through simple filtration under slightly reduced pressure. Interestingly, the prepared nanoporous membrane not only maintains the regular porosity, but also stands without supporting. More significantly, the membrane can realize the pinpoint size-selective separation of semiconductor quantum dots (QDs) in accuracy of 0.1 nm, just encountering a quick filtration under reduced pressure.

## Results

### Construction of bridging stick with bulky cationic heads

**Figure 1:**
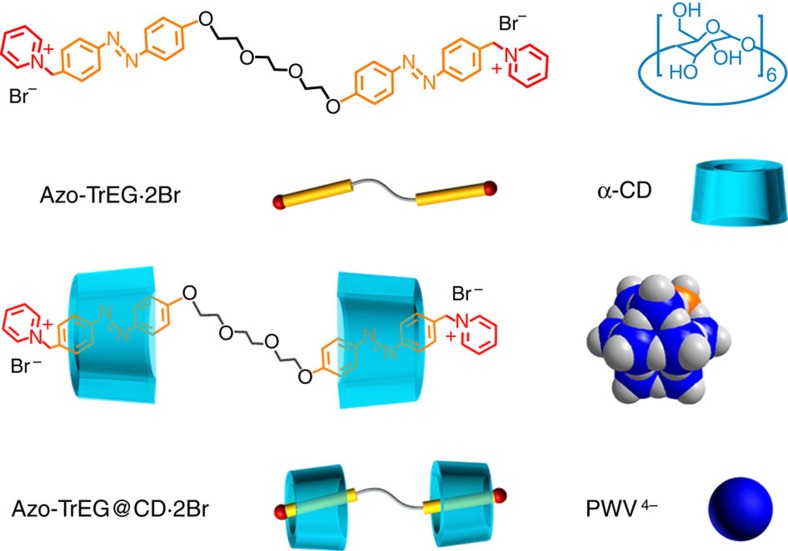
Schematic representation chemical structures and representations. Cationic bolaform molecule bearing two guest groups (Azo-TrEG·2Br), non-ionic host molecule (α-CD), pseudorotaxane unit (Azo-TrEG@CD·2Br) and POM polyanionic cluster (PWV^4−^).

**Figure 2:**
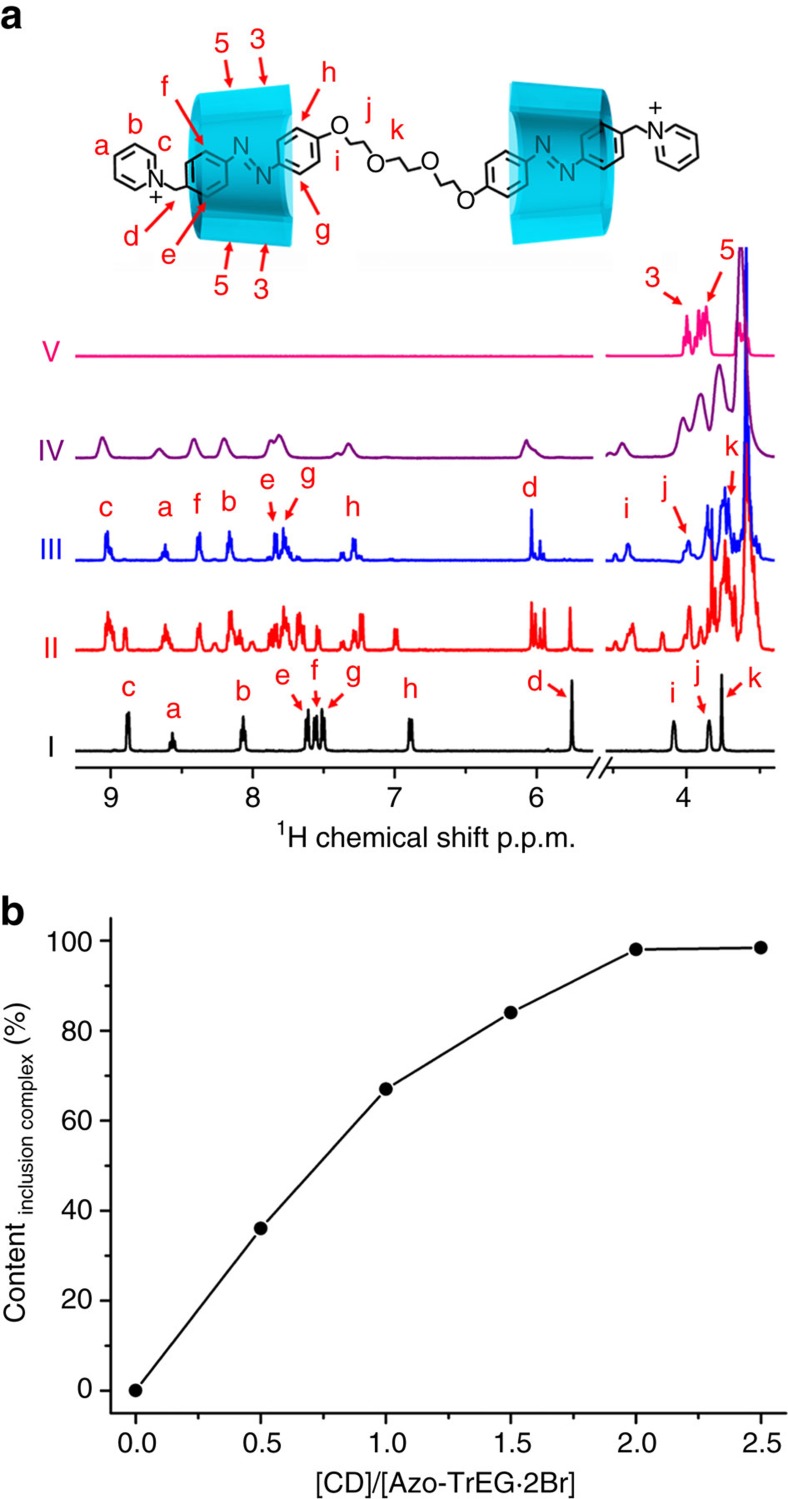
^1^H NMR spectra. (**a**) Chemical shift of Azo-TrEG·2Br upon addition of 0 (I), 1.0 (II), 2.0 (III) eq. CDs; Azo-TrEG@CD·2Br upon addition of 0.5 equivalent PWV^4−^ (IV); and isolated CD (V) (D_2_O, 25 °C), (**b**) plot of relative content of inclusion complex calculated from integral area of H(d) versus the molar ratio of CD to Azo-TrEG·2Br.

The bolaform cationic molecules (Azo-Tr/TeEG·2Br) comprised of two cationic azobenzene (Azo) groups connecting with a tri-/tetra-ethylene glycol (Tr/TeEG) spacer were synthesized and their chemical structures were characterized by ^1^H NMR and electrospray ionization mass spectra (ESI-MS) (Supplementary Figs 1−10). The reasons we designed such a stick molecule are that the two cationic heads on both sides can perform as a stick to bind with polyanions (Fig. 1), and the Azo group can recognize with CD to block the possible aggregation and control the steric adaptation. The spacer is used to adjust the pore size and flexibility of as-prepared self-assemblies. We firstly examined host–guest interaction between Azo-TrEG·2Br and CD by ^1^H NMR spectra in D_2_O (Fig. 2, Supplementary Fig. 11 and Supplementary Table 1). Upon addition of host CDs, all proton signals of guest bolaform Azo-TrEG·2Br shift downfield. The largest shift (0.82 p.p.m.) for proton H(f) in Azo group is observed, while the signals of H(j) and H(k) belonging to spacer chain shift downfield (0.14 and 0.07  p.p.m.) slightly, indicating that only Azo groups are included in CDs. The plot of inclusion amount calculated by relative integral area of H(d) versus the molar ratio of CD to Azo-TrEG·2Br suggests that over 96% Azo-TrEG·2Br form pseudorotaxane in 2:1 stoichiometry (Azo-TrEG@CD·2Br). The 2D NOESY spectrum of CD and Azo-TrEG·2Br mixture (molar ratio 2:1) in D_2_O (Supplementary Fig. 12a) displays four NOE correlation signals between H(3) and H(5) in CD cavity and H(e–g) in the Azo group. Specifically, proton H(3) correlates with H(f) and H(g) at (8.37, 3.75  p.p.m.) and (7.78, 3.75  p.p.m.), while proton H(5) associates with H(e) and H(f) at (7.84, 3.58  p.p.m.) and (8.37, 3.58  p.p.m.), further identifying that Azo group is included in CD in a slightly acclivitous orientation. Moreover, the ESI-MS of CD and Azo-TrEG·2Br mixture clearly reveals formation of Azo-TrEG@CD·2Br as a [2+1] inclusion complex (Supplementary Fig. 13).

### End-capping and crosslinking reaction by ionic bond

Then, upon the addition of 0.5 equivalent of PWV^4−^ into the Azo-TrEG@CD·2Br solution, the cationic heads of the bridging stick linker can be tethered by polyanionic cluster capper via electrostatic interaction, yielding an organic–inorganic supramolecular rotaxane through electrostatic interaction. By referring to the chemical shifts of isolated Azo-TrEG@CD·2Br, all proton signals of pyridinium head group broaden and shift downfield, for instance of 0.05  p.p.m. movement for H(c) shown in Fig. 2a, implying the braced electrostatic interaction. The X-ray photoelectron spectra (Supplementary Fig. 14) show that the counterions of Azo-TrEG@CD·2Br have been substituted by PWV^4−^ cluster in the final complex after separation from solution. Combined these results with elemental analysis (Supplementary Table 2), a full charge neutralization between Azo-TrEG@CD^2+^ and PWV^4−^ is affirmed, thus yielding a tightly bound electrostatic complex with Azo-TrEG@CD^2+^ units. ^31^P NMR spectra of PWV^4−^ (Supplementary Fig. 15) show no change in chemical shift after the substitution of counterions, implying the stable cluster's structure in [Azo-TrEG@CD][PWV]. Meanwhile, the proton signals of Azo groups still closing to their included state support the inclusion interaction maintaining in the terminal locked polyrotaxane. However, compared with Azo-TrEG@CD·2Br alone, only three NOE correlation signals appear in 2D NOESY NMR spectrum of [Azo-TrEG@CD][PWV] (Supplementary Fig. 12b). The proton correlations of H(e) with H(5), and H(g) with H(3) retain constant. Instead of correlations of H(f) with H(3) and H(5), a new signal at (6.07, 3.62  p.p.m.) that is assigned to the correlation of H(d) and H(5) appears. These definite correlations reveal that the electrostatic combination to the cationic head results in a large tilting of Azo group in CD cavity, which compresses the outside methylene group next to pyridyl head deeply into CD cavity. The tilting angle corresponding to the normal of CD cavity is estimated to be around 23.4° (Supplementary Fig. 16). Interestingly, from proton chemical shifts of CD molecule after mixing with PWV^4−^ (Supplementary Fig. 17), we observe the interaction between the narrow ring of CD and PWV^4−^ existing in aqueous solution. Therefore, the acclivitous recognition model is conducive to decrease the distance and strengthen the interaction between the narrow ring of CD and cluster. The crystal structure analysis on the interaction between CD and a similar POM to the present one in the recent reported result highly supports this assignment^44^.

**Figure 3:**
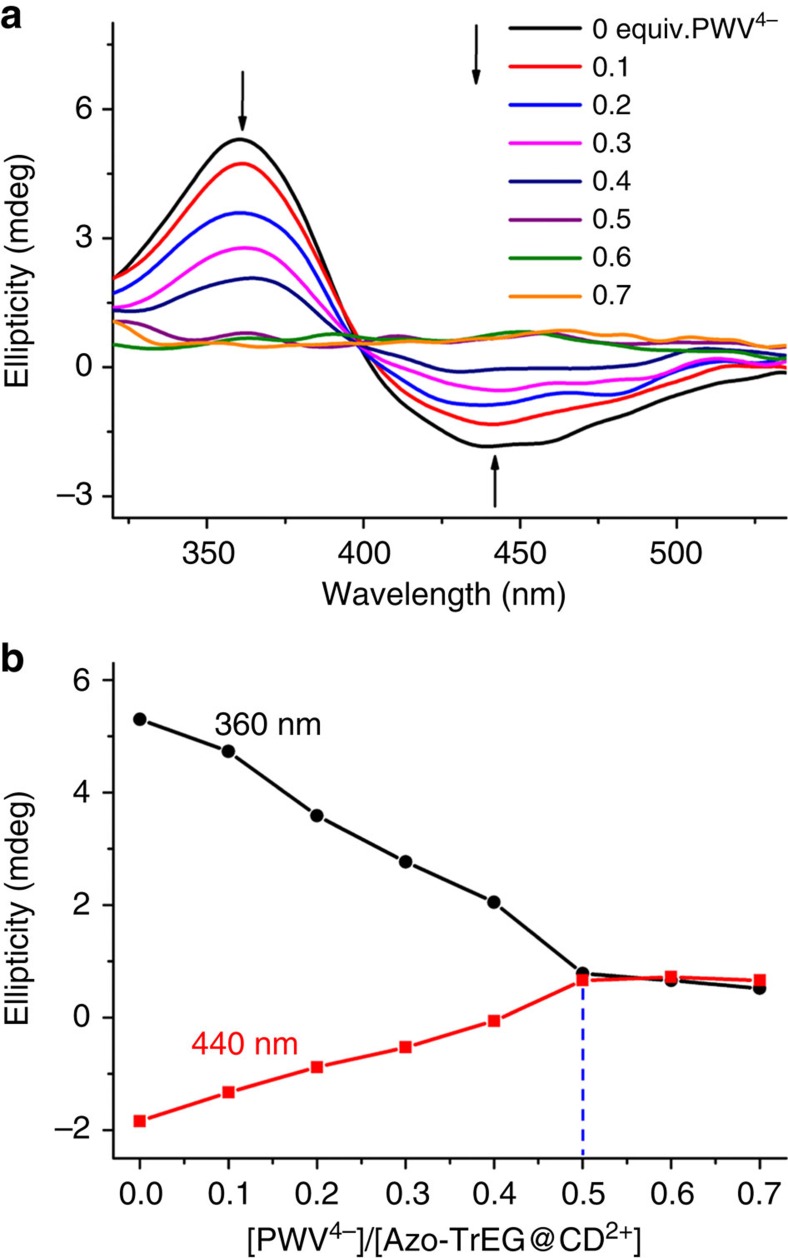
Circular dichroism spectra (CDS). (**a**) Azo-TrEG@CD·2Br aqueous solution upon addition of PWV^4−^ (from 0 to 0.7 equivalents), and (**b**) intensity plots of Cotton signals at 360 and 440 nm versus the molar ratio of PWV^4−^ to Azo-TrEG@CD^2+^.

We further used circular dichroism spectroscopy to characterize inclusion interaction in POM-locked polyrotaxane. The induced spectrum of isolate Azo-TrEG@CD·2Br (Fig. 3a) shows a positive Cotton effect corresponding to π−π* transition of Azo group at 360 nm and a negative Cotton effect ascribing to n−π* transition of the same group at 440 nm, indicating the strong inclusion interaction. The spectral feature indicates Azo group in approximately parallels to the normal of CD cavity^45^,^46^. Upon addition of PWV^4−^ (0.1–0.7 equivalent), both intensities of positive and negative Cotton effects gradually decrease. As is known, the deviation of transition moment for guest molecule from normal axis of CD cavity leads to the weakness of induced chirality until to silence^46^,^47^. The acclivitous state of Azo group in CD cavity further certifies its source from electrostatic interaction, which is in good accordance with the preponderant mode inferred from 2D NOESY spectrum. Thus, the model can be used to trace the binding ratio between PWV^4−^ cluster and Azo-TrEG@CD^2+^ by simply monitoring induced Cotton signals of Azo groups. The intensity plots of Cotton effects at 360 and 440 nm versus the molar ratio of PWV^4−^ to Azo-TrEG@CD^2+^ illustrate a definite 1:2 stoichiometry at the turning point when the charges of PWV^4−^ are fully neutralized by Azo-TrEG@CD^2+^ (Fig. 3b). Considering the multiple binding sites in above building components, a supramolecular ionic framework becomes apparently dominant in limited possibilities of connection geometry.

### Synergistic ionic self-assembly for 2D framework

In an attempt to assemble 2D frameworks by using the present system via ionic interaction, steric effect and other additional interaction, such as hydrogen bond, must be combined. In other case of POM-based ionic self-assembly system, the negative charges of POMs are widely accepted to be delocalized though there was no a precisely theoretical calculation due to the difficulty for solution system^41^. As an indirect example, the DFT calculation indicates that the electrons move towards contacting site when a POM adsorbs on graphene surface^42^. The molecular dynamics simulation for POMs in aqueous solution revealed that the paired ions moved freely within the region bounded by Bjerrum's length^43^. Thus, the delocalized negative charges on a POM catch its counterions anywhere around the cluster surface. But the distribution of negative charges corresponding to binding sites is affected or dominated by steric effect and additional interaction among those trapped cationic components dynamically. As an example, the lateral van der Waals interaction was found to trigger the cationic groups around each POM from a mean distribution to a separated state of hydrophilic and hydrophobic components. This means that organic cations accumulate on opposite sides or even equatorial plane of one POM, depending on the packing fashion propelled by the interfacial energy^48^,^49^,^50^,^51^,^52^. Therefore, under driven by proper addition interaction and steric effect, the preferential distribution of negative charges on POM, directed by counterions, becomes possible.

In light of this, PWV^4−^ cluster performing the node in construction of 2D IOIF upon simply modulating binding angle between cationic bridging sticks surrounding it becomes rational. Based on similar understanding, the non-centrosymmetric PWV^4−^ cluster does not affect the movement of negative charges since those self-assembled structures of POM complexes in solutions were proved to be independent on the symmetry of POM clusters^53^. As for detailed estimation on steric adaptation between CD-shielded cations and PWV^4−^ cluster, firstly, the POM has enough space to accommodate four CD-shielded cations packing in one plane, which is the prerequisite for 2D framework (complemented structural analysis shown in Supplementary Figs 18,19). Secondly, the calculated distance between neighbouring CD shields in a tetragonal fashion is ∼0.23 nm (Supplementary Fig. 19b), in perfect agreement with the reported distance of hydrogen bond^54^,^55^, indicating the favourable lateral interaction between the narrow rings of neighbouring CD-shielded cations. Of course, besides the planar square framework, the steric tetrahedron binding style is also possible theoretically. However, suppose the tetrahedron structure was preferential, the distance between CD shields would increase to ∼0.58 nm (Supplementary Fig. 20), too far for the formation of hydrogen bonding between neighbouring CD shields. So, the efficient hydrogen bonds of CD-shielded cations around each POM assists for the 2D framework.

### Characterizations for 2D IOIF structure

**Figure 4:**
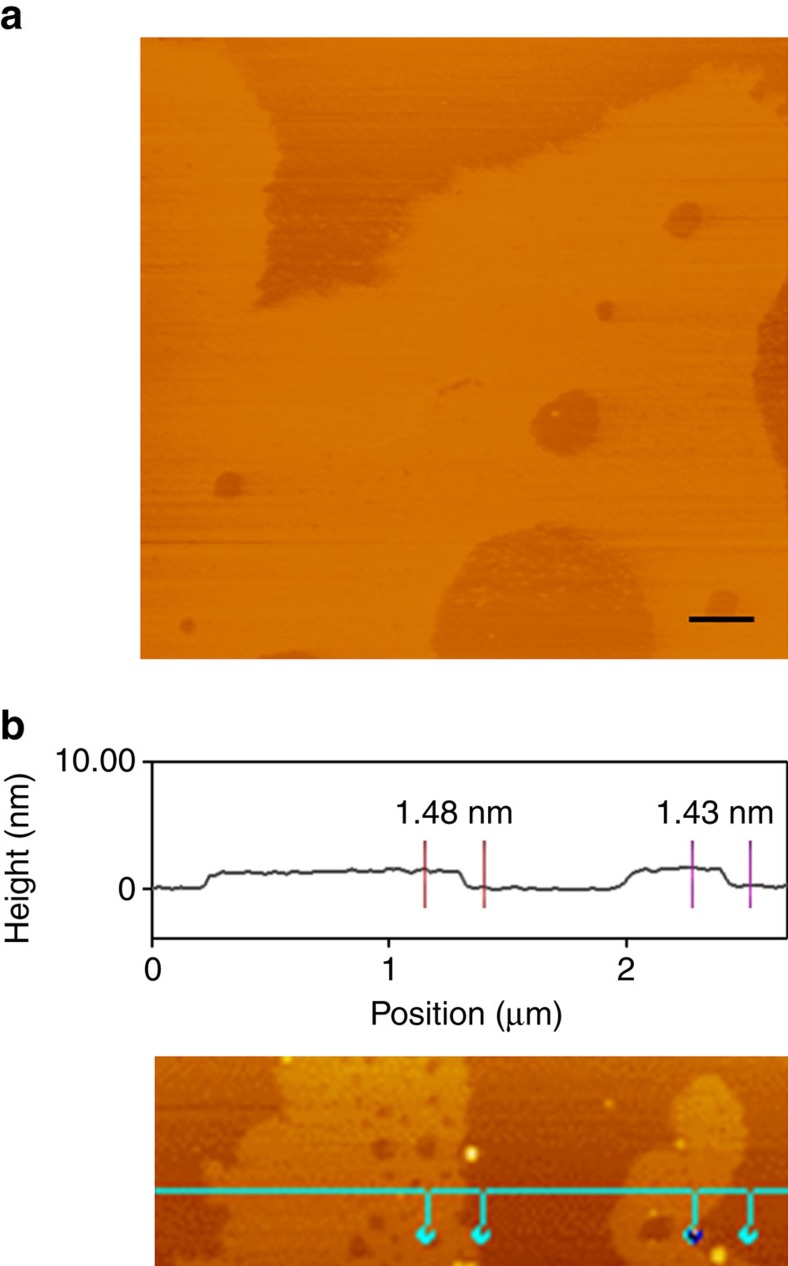
AFM image. (**a**) Tapping mode image and (**b**) height profile analysis of [Azo-TrEG@CD][PWV] architecture spreading on mica. Scale bar, 500 nm (**a**).

Neither Azo-TrEG@CD·2Br nor PWV^4−^ alone shows Tyndall phenomenon in aqueous solution, while a distinct light-scattering effect emerges in their 2:1 mixture solution (Supplementary Fig. 21), implying the generation of ionic self-assembly. We further characterized the structure by using microscopic techniques. Atomic force microscopic (AFM) image demonstrates that the supramolecular architectures appear as very thin sheets with irregular shape at the beginning (Supplementary Fig. 22). After a while, micrometre-scale sheets can be observed (Fig. 4) but the thickness maintains constant, indicating stepwise 2D growing of the architectures. Based on the thickness analysis in a large area, the average height is at 1.43−1.48 nm, in perfect agreement with the interlayer spacing (1.49 nm) measured by powder XRD of freeze-dried sample (Supplementary Fig. 23a). Considering the accord of this value with the diameter (1.46 nm) of CD ring^56^, a single-layer 2D assembling structure can be rationally inferred.

**Figure 5:**
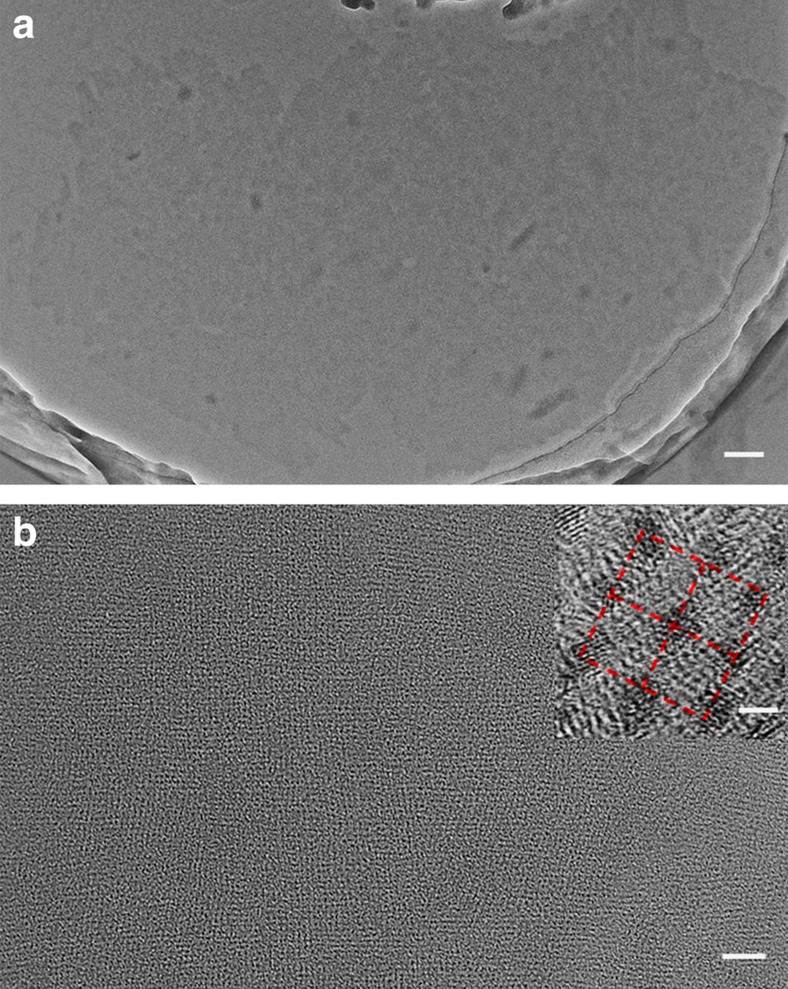
TEM images. Single-layer self-assembly of [Azo-TrEG@CD][PWV] IOIF in: (**a**) wide and (**b**) amplified scale, while the insert presents a high resolution TEM image taken from **b**. Scale bars, 100 nm (**a**) 20 nm (**b**) 2 nm (inset in **b**).

**Figure 6:**
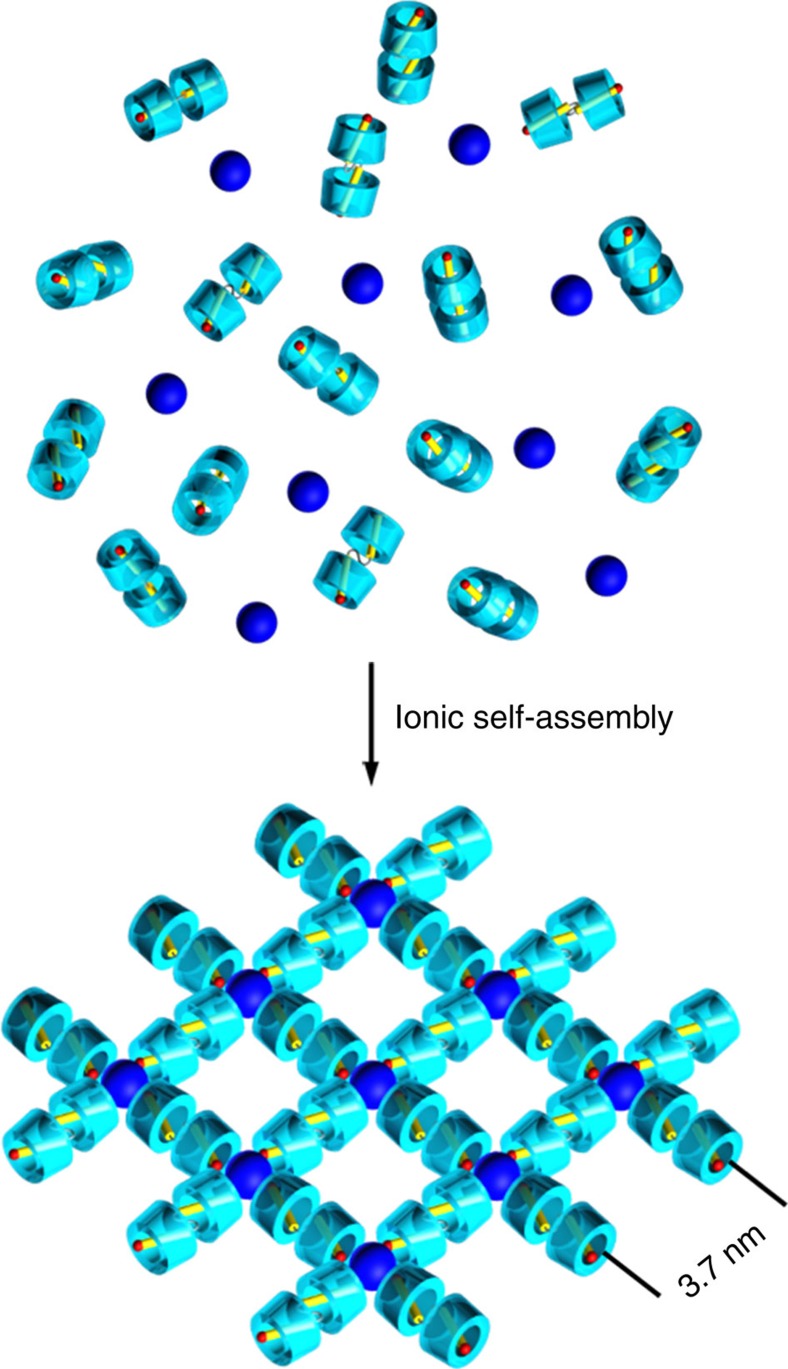
Schematic representation of mechanism. Possible process for spontaneous formation of 2D supramolecular framework via ionic self-assembly of cationic pseudorotaxane unit (Azo-TrEG@CD^2+^) and POM cluster (PWV^4−^).

Transmission electron microscopic (TEM) measurements further demonstrate the micrometre-scale sheets of singly layered ionic supramolecular assembly (Fig. 5a). The wrinkled and folded edges (Supplementary Fig. 24) illustrate the high flexibility and free-standing feature of the single-layer assembly. The energy-dispersive X-ray spectral analysis of the observed sheets points out the presence of tungsten element (Supplementary Fig. 25), confirming the inorganic clusters existing in the sheets. Because of the electron density contrast between organic and inorganic components, in the case without addition of any staining agents, the inorganic clusters can be well discerned as dark spots locating at the nodal points. The formed long-range uniform orthogonal mesh structure with edge length ∼3.7 nm in the single-layer sheet is observed definitely (Fig. 5b). This fantastic framework structure is further identified by XRD measurement (Supplementary Fig. 23b and Supplementary Table 3). The diffraction pattern of sample film prepared by filtration of IOIF self-assembly solution exhibits nine diffraction peaks, which can be perfectly indexed into an in-layer lamellar structure with 3.7 nm of spacing, in perfect accord with the value estimated from high resolution TEM image. This value is also in good agreement with the ideal length calculated from planar square framework (3.8–4.6 nm) after considering the utmost shrinkage and stretching of TrEG spacer inserted in bolaform cation (Supplementary Fig. 26). These results strongly point out that the square framework exists in the single-layer 2D architecture comprised of Azo-TrEG@CD^2+^ bridging stick and PWV^4−^ node via electrostatic interaction, as illustrated in Fig. 6. It is worth pointing out that the size heterogeneity of PWV^4−^ and Azo-TrEG@CD^2+^ and the flexibility of IOIF can weaken the interlayer interactions, thus leading to dominant single-layer frameworks in water. Besides, the hydrophilicity of the building blocks facilitates the stable dispersion of single-layer IOIF in water.

To demonstrate the role of α-CD in synergistic ionic self-assembly for 2D framework structure, we repeat the preparation of single layered self-assembly by using β-CD to replace α-CD. As predicted, we do not observe any expected layer assemblies, but instead, nubbly and distorted bulk aggregations are found (Supplementary Fig. 27). This difference supports above analysis that the size matching and the lateral interaction between neighbouring CD molecules drives the tetragonal distribution of α-CD-shielded cations around POM. Because the larger size of β-CD, the used POM cannot provide enough space for four β-CD-shielded cations packing in the same planar style (Supplementary Fig. 28) as that of α-CD. Consequently, a steric distribution becomes unavoidable.

### Characterizations and nano-size separation of IOIF membrane

**Figure 7:**
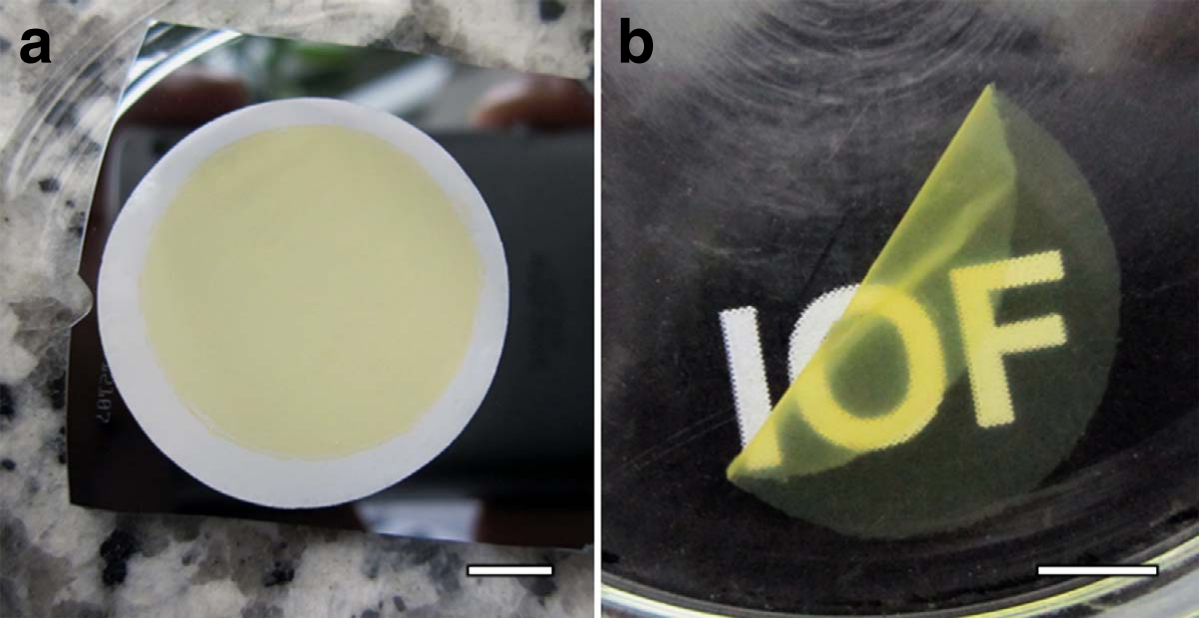
Digital photographs. (**a**) The as-prepared IOIF membrane on polycarbonate filter and (**b**) the isolated IOIF membrane obtained through drying in oven at 40 °C for 48 h and dissolving polycarbonate supporting filter in chloroform. Scale bars, 0.5 cm (**a**,**b**).

The uniform mesh-like structure and the flexibility of the single-layer frameworks in aqueous solution exhibit the processing feature and simplify the fabrication of nanoporous membrane to a facile suction filtration under a slightly reduced pressure. Typically, the Tr-membrane (0.25 mg cm^−2^, 2 cm in diameter) was prepared from filtration of [Azo-TrEG@CD][PWV] solution (0.04 mg ml^−1^, 20 ml) on a supporting polycarbonate filter under the vacuum pressure of −2,000 Pa, as shown in Fig. 7a. After drying at 40 °C for 48 h and suffering the dissolution of polycarbonate filter in chloroform, a free-standing transparent IOIF membrane is obtained with rough thickness ∼0.43 μm (Fig. 7b and Supplementary Fig. 29b). No obvious defects or pinholes (Supplementary Fig. 30) have been found, displaying intactness and good mechanical stability. With this method, in general, the area of the IOIF membranes is not restricted and the membranes can be prepared independent of supporting substrates' shape and size. Considering the torsion-induced shrinking of TrEG chain, the possible mesh size for the prepared Tr-membranes is estimated in the region from 2.4 to 3.4 nm (Supplementary Fig. 31). These specific features including the regular porosity and flexibility, especially the convenience in preparation provide applicable separation capability for bigger size objects that general MOF materials could not conduct.

To demonstrate the capacity of the supramolecular IOIF in size-selective separation, three kinds of small molecules, rhodamine B, xylenol orange (pH=4.0 and 7.9) and the mixture of α-, β- and γ-CDs, which are positive, negative and nonionic at different pH conditions while maintaining the size less than 2 nm, were chosen as filtered chemical objects. The rhodamine B and xylenol orange filtrates were detected by UV–vis spectra (Supplementary Fig. 32), and CD mixture filtrate was detected by matrix-assisted laser desorption/ionization time-of-flight (MALDI-TOF) mass spectra (Supplementary Fig. 33). As predicted, all three kinds of small molecules, regardless of positive charge, negative charge or non-charge, can pass through the membrane without obvious quantity loss. According to the size matching, the result indicates a fluently passing ability for organic molecules smaller than the diameter of estimated square pores.

**Figure 8:**
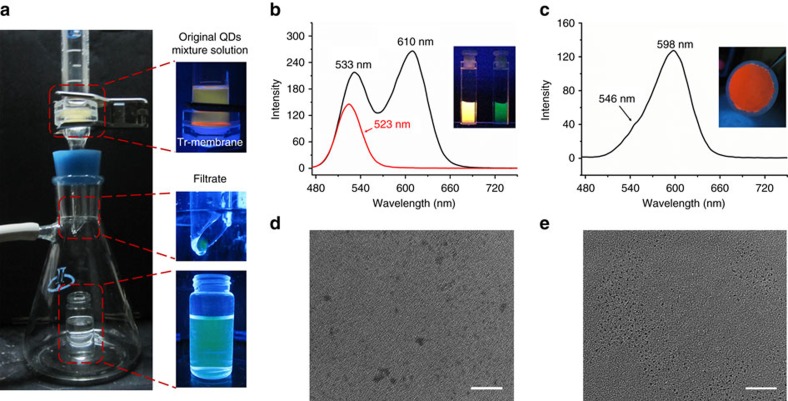
Precise nano-size-selective separation. (**a**) Digital photographs of installation and corresponding amplifications of main parts under 365-nm light irradiation for examination of QDs mixture solution of TG-1 (*λ*_max_=533 nm and *D*=3.3 nm) and TG-2 (*λ*_max_=611 nm and *D*=4.4 nm) passing through Tr-membrane. Luminescent spectra of (**b**) initial QDs mixture solution (black line) and filtrate (red line), where the inset presents corresponding photographs of original solution (left) and filtrate (right) under 365-nm light; (**c**) residual QDs washed out from filtrated Tr-membrane, where the inset is the corresponding photograph of residual QDs under 365-nm light. TEM images of QDs from (**d**) initial mixture solution and (**e**) filtrate. Scale bars, 20 nm (**d**,**e**).

QDs are one of typical competitive luminescent probes for bioimaging and emitting materials for illumination and display^57^. Because the emission properties of QDs are highly dependent on size and distribution, the screen and separation by sizes from the ensembles are significant for further functional optimization. The capability of prepared IOIF membrane for the separation of QDs is evaluated. Two differently sized CdTe QDs modified with 1-thioglycerol (TG) in aqueous solution are applied. The smaller one (TG-1) has an average particle size *D*=3.3 nm in diameter with green emission (*λ*_max_=533 nm) and the bigger one (TG-2) has the size *D*=4.4 nm in diameter with red emission (*λ*_max_=611 nm) after considering the thickness of modified surface layer. The mixture solution of TG-1 and TG-2 was filtered through Tr-membrane and the filtrate was monitored by emission spectrum. Interestingly, the luminescent photographs (Fig. 8a,b, inset) using to monitor the filtration process show that the orange luminescence of QDs mixture solution turns green after the filtration, indicating that only smaller size QDs passed through the Tr-membrane. Accompanying by a 10 nm blue-shifting (*λ*_max_=523 nm) of the emission band of the filtrate, the full-width at half-maximum (FWHM) narrows from 41.4 to 39.1 nm. Apparently, this spectral change corresponds to particle size having decreased to ∼3.0 nm, while the larger size part of TG-1 and whole TG-2 are blocked by the supramolecular polymer membrane, as supported by red luminescence on filtered membrane (Fig. 8a,c, inset). By washing the filtered membrane with water, the collected residue QDs display two emission bands at 546 and 598 nm, further confirming that the larger sized part of TG-1 and whole TG-2 in QD ensembles have been successfully separated off. We also characterized the QDs in filtrate by TEM and indeed observed QDs with smaller and narrower size distribution than those without encountering filtration (Fig. 8d,e). These results verify that the critical separation size is in 3.0−3.3 nm, very close to the estimated mesh dimension in prepared Tr-membrane. After drying at 40 °C for 48 h, the Tr-membrane has the same filtration capability as the fresh made one (Supplementary Fig. 34), indicating the maintained mesh structures and IOIF membrane strength after drying treatment.

### Mesh-size modulation and separation efficiency

**Figure 9:**
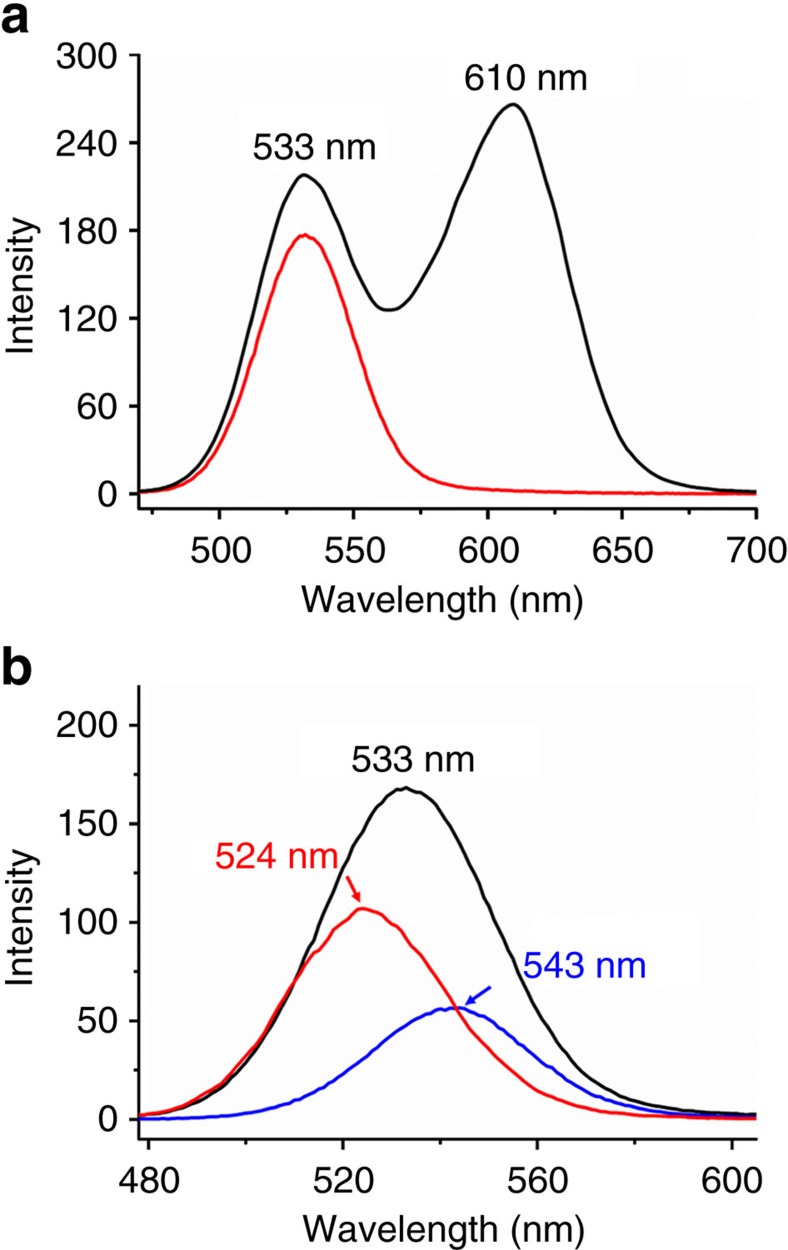
Two-step separation. Fluorescent spectra of (**a**) initial QDs mixture solution of TG-1 (*λ*_max_=533 nm, *D*=3.3 nm) and TG-2 (*λ*_max_=611 nm, *D*=4.4 nm) (black line), and the filtrate (red line) passing through Te-membrane; and (**b**) the filtrate obtained from the first filtration of original TG-1 and TG-2mixture solution through Te-membrane (*λ*_max_=533 nm, black line), the filtrate obtained from the second filtration through Tr-membrane (*λ*_max_=524 nm, red line), and the residual QDs washed out from the filtrated Tr-membrane (*λ*_max_=543 nm, blue line).

The pore of prepared single-layer framework is adjustable and can be modulated by simply increasing the length of flexible spacer: TeEG. The obtained [Azo-TeEG@CD][PWV] displays the whole characteristics of [Azo-TrEG@CD][PWV] on framework structure and filtration property except the increased pore size (2.4−4.1 nm) after considering the shrinkage and stretching of TeEG chain (Supplementary Figs 35–41 and Supplementary Table 2). The TG-1 passes through Te-membrane in both cases of isolated solution (Supplementary Fig. 42) and its mixture with TG-2 (Fig. 9a). However, TG-2 is still impossible to penetrate through the mesh of Te-membrane. The critical value estimated from the filtration cutoff for the Te-membrane should be in the region between sizes of TG-1 and TG-2. More interestingly, we can combine the two IOIF membranes bearing different mesh dimensions for selective size optimization of QDs mixture. For example, for the mixture solution of TG-1 and TG-2, we firstly filtered out the larger sized TG-2 by using Te-membrane, and then conducted another filtration of the filtrate by using Tr-membrane. Thus, the QDs showing luminescence at 543 nm with the dimension between two cutoff sizes of Tr- and Te-membranes are obtained by washing the residue on Tr-membrane (Fig. 9b). This simple combination of filtration membranes provides more precise classification of QDs. By taking isolated TG-1 as reference, the separation experiment indicates that the smaller size part of TG-1 can also be further separated by using Tr-membrane (Supplementary Fig. 43). The spectral analysis of the residual QDs blocked by Tr-membrane indicates the larger-size composition of TG-1 in diameter of 3.6 nm (*λ*_max_=542 nm). With this fast and facile filtration, we provide an alternative analysis method for size distribution of QDs in addition to high-resolution TEM.

Generally, the aforementioned separation is independent of the surface species stabilizing QDs because when 3-mercaptopropionic acid-modified QDs in size of 4.0 (MPA-1) and 4.8 nm (MPA-2) are used in the filtration, we also obtain identical separation effect (Supplementary Figs 44–46). By evaluating the filtration of MPA-1 and its mixture of TG-2 with Te-membrane, we can definitely deduce the practical critical value of separation in a much precise region of 3.9−4.0 nm (Supplementary Fig. 45).

The separation efficiency of QDs is carried out by evaluating relative fluorescence intensity before and after the filtration. As shown in Table 1, for Te-membrane, because the size of TG-1 (3.3 nm) is much smaller than the mesh size of Te-membrane, up to 93.4% of TG-1 pass through the filter (Supplementary Fig. 42). Considering few larger sized QDs having been blocked on the IOIF membrane, the filtration displays very high efficiency for QDs in smaller size. In the case of mixture of TG-1 and TG-2, because the TG-2 in larger size are impossible to pass through the filter, the residue attached on the membrane obstructs the passing channel of TG-1, generating a decreased separation efficiency to 81.3% (Fig. 9a). For Tr-membrane, only the smaller size part of TG-1 penetrate through the membrane, leading to decreased separation efficiency of 76.3% and 73.4% for isolated TG-1 (Supplementary Fig. 43a) and its mixture with TG-2 (Fig. 8b), respectively. For other separations, Te-membrane performs 80.4% separation efficiency for MPA-1 alone (Supplementary Fig. 45a) and 71.2% for the mixture of MPA-1 and TG-2 (Supplementary Fig. 45b), while Tr-membrane displays 68.8% separation efficiency for TG-1 mixing with MPA-2 (Supplementary Fig. 46b). Because of the emission of MPA-1 locating at 523 nm, it is difficult to evaluate the separation efficiency of Tr-membrane for TG-1 and MPA-1 mixture accurately by analysing relative fluorescence intensity at the same wavelength before and after filtration (Supplementary Fig. 46a). In general, the separation efficiency is highly affected by the thickness of IOIF membrane. We prepared Tr-membranes with different thickness by varying the concentration of sample solution (Supplementary Fig. 29). With the concentration decreasing, the thickness becomes thinner and uneven. When the thickness is less than 200 nm, the separation of QDs mixture becomes incomplete because of the possible defects in the membrane formation upon suction, which results in leakage of some larger sized QDs into the filtrate. But, with the thickness increasing, the separation of QDs mixture solution performs well. The emission band and FWHM in the spectra of filtrates show no obvious change except a gradual decrease of separation efficiency (Supplementary Fig. 47).

### Durability and reusability

To demonstrate the durability of prepared IOIF membranes, 20 time-separation experiments (20 ml for each separation, 400 ml in total) for TG-1 and TG-2 mixture solution were consecutively carried out by using the same Tr-membrane. After the continuous separation, a slight decrease of separation efficiency is found but there is no significant change by examining the emission band and FWHM of the filtrate (Supplementary Fig. 48 and Supplementary Table 4), implying the good durability of the IOIF membranes. The burst strength of Tr-membrane (0.25 mg cm^−2^, ∼0.43 μm) spreading on a support bearing average pore of 220 nm in diameter is 1.49∼1.66 MPa with the test area of 3.8 × 10^−6^ cm^2^ (Supplementary Figs 49,50), revealing high stability of the IOIF membranes. Furthermore, after washing out the residual QDs, we re-dispersed the filtered Tr-membrane in water. After sonication for a while (Supplementary Fig. 51), a new Tr-membrane can be prepared by filtrating the re-dispersed solution and used to separate QDs mixture solution of TG-1 and TG-2. A similar separation capability as that of fresh one is observed (Supplementary Fig. 52), demonstrating the reusability of the IOIF membranes.

## Discussion

Overall, we have created a facile and convenient strategy for the fabrication of free-standing single-layer 2D IOIFs. In contrast to the known driving forces, electrostatic interaction was employed as the main binding force for the supramolecular hybrid frameworks. Through the synergetic ionic self-assembly of cationic pseudorotaxane unit as bridging stick and anionic PWV^4−^ as capper and node in aqueous solution, the obtained IOIF supramolecular polymer effectively overcomes the interlayer interaction and structure stiffness. The prepared single-layer framework not only integrates inorganic clusters and rotaxane-type units into branched polymeric architectures but also possesses highly ordered nanoporous mesh and good solution processability. This type of soft framework structure constructed by ionic bonding without preferential direction and its distinctive properties such as stability and plasticity offer an unprecedented opportunity to fabricate ultrafiltration membrane towards precisely screening nanoparticles. Because of the facile methodology and uniform nanoporous structure, the membranes prepared with 2D IOIF assemblies hold steady promise for practical applications in selective transport, molecular separation and dialysis systems. Meanwhile, the synergetic ionic self-assembly strategy can be hopefully extended to diverse IOIFs for advanced supramolecular materials.

## Methods

### Materials and instruments

The general chemicals, α-, β-, γ-CD (α-, β-, γ-CD), rhodamine B and xylenol orange, are the products of TCI Chemicals (China) Pvt. Ltd. Other chemicals and solvents were purchased from Beijing Chemical Reagent Industry and used as received. Acetonitrile was dried over P_2_O_5_ and distilled prior to use. N,N-dimethylformamide (DMF) was dried with CaH_2_ for several days and distilled before using. Doubly distilled water (Milli-Pore 18.2 MΩ cm^−1^) was used in the experiment. The polycarbonate membranes with 0.2-μm pore size are the product of Whatman Filters (a GE Healthcare brand). The polyanionic cluster PWV^4−^ was prepared following the published procedure^58^. Four kinds of CdTe QDs were prepared and characterized following the published procedures^59^.

^1^H NMR spectra were recorded on a Bruker AVANCE 500 MHz spectrometer while the chemical shifts were corrected by the solvent value (*δ*=4.79 p.p.m. for D_2_O and *δ*=2.50 p.p.m. for DMSO-*d*_6_). UV–vis spectra were carried out on a spectrometer (Varian CARY 50 Probe). The fluorescent spectra were carried out using the spectrophotometer (Shimadzu RF-5301PC). Circular dichroism spectroscopy were performed on a Bio-Logic MOS-450 spectropolarimeter in water with step size of 1 nm and speed of 5 nm s^−1^ at 25 °C. AFM images were taken with a SPA-300HV (Seiko, Japan) under ambient conditions. AFM was operated in the tapping mode with an optical readout using Si cantilevers. Scanning electron microscope images were acquired on a JEOL FESEM 6700F field emission scanning electron microscope (JEOL, Japan). TEM and HRTEM were conducted on a JEOL JEM 2010 electron microscope under an accelerating voltage of 200 kV without staining and energy-dispersive X-ray spectrum was collected on a FEI Tecnai F20 microscope operating at an accelerating voltage of 200 kV. XRD data were recorded on a Rigaku SmartLab X-ray diffractometer using Cu Kα_1_ radiation at wavelength of 1.542 Å. MALDI-TOF mass spectra was recorded on an autoflex MALDI-TOF/TOF (Bruker, Germany) mass spectrometer equipped with a nitrogen laser (337 nm, 3 ns pulse). The ESI-MS/MS spectra were carried out by POEMS inductively coupled plasma mass spectrometer (TJA, USA). X-ray photoelectron spectra was carried out on an ESCALAB 250 spectrometer with a monochromic X-ray source (Al Kα line, 1,486.6 eV) and the charging shift was corrected by the binding energy of C(1 s) at 284.6 eV. Inductively coupled plasma atomic emission spectrometry (ICP-AES) was carried out on the PerkinElmer Optima 3300DV (PerkinElmer, USA). Organic element analysis was carried out on the vario MACRO cube CHNS (Elementar, Germany).

### Synthesis of Azo-Tr/TeEG·2Br

The bolaform cationic organic compounds were synthesized according to the route in Supplementary Fig. 1. Azo compound **a**, sulfonated compound of glycol **b**, coupling product **c** was prepared according to the literatures^60^,^61^. Compound **c** bearing TrEG or TeEG chain (1 g, 1.44 or 1.35 mmol) was added to a 10 ml round bottom flask containing 5 ml of DMF and 2 ml of pyridine. The reaction mixture was heated under stirring in an oil bath at 100 °C for 48 h. Then the reaction solution was cooled to room temperature and transferred dropwise to 500 ml of ethyl acetate. The yielded precipitate was collected by filtration and washed with ethyl acetate (1,000 ml) for three times to give the product (0.93 g, 76% for Azo-TrEG·2Br; 0.90 g, 74% for Azo-TeEG·2Br). ^1^H NMR (500 MHz, D_2_O) for Azo-TrEG·2Br: *δ* 8.87  p.p.m. (d, *J*=5.6 Hz, 4H, Ar-H), 8.57 (t, *J*=7.9 Hz, 2H, Ar-H), 8.07 (t, 4H, Ar-H), 7.62 (d, *J*=8.4 Hz, 4H, Ar-H), 7.57 (d, *J*=9.0 Hz, 4H, Ar-H), 7.51 (d, *J*=8.5 Hz, 4H, Ar-H), 6.92 (d, *J*=9.0 Hz, 4H, Ar-H), 5.75 (s, 4H, CH_2_), 4.10 (t, 4H, CH_2_), 3.86 (t, 4H, CH_2_) and 3.77 (s, 4H, CH_2_). ^13^C NMR (125 MHz, DMSO-*d*_6_): *δ* 161.7, 152.8, 146.3, 146.2, 145.0, 136.4, 130.1, 128.7, 124.9, 123.0, 115.3, 70.1, 68.9, 67.8 and 62.9. ESI-MS (*m*/*z*) [M]^2+^: calculated for C_42_H_42_N_6_O_4_: 347.4; found: 347.4. ^1^H NMR (500 MHz, DMSO-*d*_6_) for Azo-TeEG·2Br: *δ* 9.26  p.p.m. (d, *J*=5.7 Hz, 4H, Ar-H), 8.67 (t, *J*=7.8 Hz, 2H, Ar-H), 8.22 (t, *J*=7.1 Hz, 4H, Ar-H), 7.98−7.80 (m, 8H, Ar-H), 7.72 (d, *J*=8.3 Hz, 4H, Ar-H), 7.17 (d, *J*=8.9 Hz, 4H, Ar-H), 5.97 (s, 4H, CH_2_), 4.21 (t, 4H, CH_2_), 3.79 (t, 4H, CH_2_) and 3.65−3.50 (m, 8H, CH_2_). ^13^C NMR (126 MHz, DMSO-*d*_6_) *δ* 162.1, 152.8, 146.63, 145.57, 145.4, 136.8, 130.4, 129.0, 125.3, 123.3, 115.6, 70.4, 70.3, 69.3, 68.2 and 63.3. ESI-MS (*m*/*z*) [M]^2+^: calculated for C_44_H_46_N_6_O_5_: 369.2; found: 369.6.

### Preparation of Azo-Tr/TeEG@CD·2Br and [Azo-Tr/TeEG@CD][PWV]

In a typical procedure, Azo-TrEG·2Br (4.5 mg, 5.3 μmol) and α-CD (10.2 mg, 10.5 μmol) were mixed and dissolved in 60 ml water under sonication for 0.5 h to generate pseudorotaxane unit Azo-TrEG@CD·2Br. The final framework assembly of [Azo-TrEG@CD][PWV] (0.17 mg ml^−1^) was constructed by simply mixing the as-prepared Azo-TrEG@CD·2Br and PWV^4−^ solution that is prepared by dissolving PWV^4−^ (7.6 mg, 2.6 μmol) in 60 ml water according to certain stoichiometry.

### Preparation of IOIF membrane

The IOIF membranes were prepared by a simple filtration of sample solutions containing single-layer IOIF self-assemblies through a commercial filter with even dispersed pores in certain size under vacuum pressure of −2,000 Pa. After washing with water, the prepared nanofiltration membrane has the same size as the effective filtration area of supporting substrate used in the installation. In a typical procedure, 20 ml as-prepared [Azo-TrEG@CD][PWV] solution (0.04 mg ml^−1^) was filtered over a supporting filter (Whatman Nuclepore Track-Etched Polycarbonate Membrane; effective filtration area: 3.14 cm^2^; pore size: 200 nm) under the preset vacuum pressure. Water (10 ml) was subsequently used to wash the membrane. Under the same vacuum pressure, the IOIF membranes with various thicknesses were prepared by using 20 ml sample solution with concentrations: 0.02, 0.04, 0.06 and 0.08 mg ml^−1^, and corresponding thicknesses are measured to be 0.20∼0.35, 0.43, 1.43 and 2.19 μm, respectively.

### Filtration of small organic molecules

The solutions of dyes, rhodamine B and xylenol orange, were prepared at the concentration of 0.42 and 0.48 mM, respectively. In the case of CD mixture, each of concentrations of α-CD, β-CD and γ-CD was maintained at 5.1 mM. In all filtration experiments, the volume of the original solutions was 20 ml, the vacuum pressure was about −5,000 Pa, and the flux was set at 15.3 m^3^ m^−2^ h^−1^ bar^−1^.

### Size-selective separation of CdTe QDs

In all QDs filtration experiments, the volume of QD solutions for each filtration was maintained at 20 ml, the vacuum pressure was about −5,000 Pa, and the flux was ∼15.3 m^3^ m^−2^ h^−1^ bar^−1^. The concentrations of QDs were estimated as follows: 0.05 mM for TG-1, 0.10 mM for TG-2, 0.02 mM for MPA-1, 0.10 mM for MPA-2, 0.05 and 0.10 mM for the mixture of TG-1 and TG-2, 0.05 and 0.02 mM for the mixture of TG-1 and MPA-1, 0.05 and 0.10 mM for the mixture of TG-1 and MPA-2, and 0.02 and 0.10 mM for the mixture of MPA-1 and TG-2, respectively, according to their absorbance. By putting the filtered Tr-membrane into 10 ml water and undergoing an oscillation for a while, the residual QDs could be washed out from the membrane (Supplementary Fig. 51). During this process, it is hard to maintain the membrane intact. The QDs mixing with a few fragments of Tr-membrane in solution could be separated by centrifugation (1,500 r.p.m., 3 min).

### Sample preparation and measurement for TEM

We used a thin copper ring to spread a thin layer of [Azo-Tr/TeEG@CD][PWV] solution (0.02 mg ml^−1^) like a soap film and cast it on a copper grid. The procedure was repeated three times for one sample to ensure enough amounts of samples being attached on the copper grid. During the measurement, longer time exposure to the high-energy electron beam will destroy the framework structure, resulting in less ordered structure. The HRTEM images were tracked within a quite short time. In order to obtain a clear high contrast image, we used a smart camera technique for image collection, which was based on a continuous acquisition of images to get high image contrast. Because of the heating disturbance of the sample during the electron beam irradiation, the image superposition brought slight ghosting phenomenon, which causes the size of inorganic clusters seemed larger than their ideal dimension.

### Sample preparation for AFM measurement

The sample for AFM measurement was prepared by a dip-coating technique. First, we dipped a mica wafer quickly into the as-prepared [Azo-Tr/TeEG@CD][PWV] solution with the concentration of 0.02 mg ml^−1^, and then slowly withdrew at a constant speed of 1 mm min^−1^. During the process, the IOIF assembly was attached on the surface of the mica in a single layer.

### Sample preparation for XRD measurement

The powdered sample was prepared by the lyophilization of the [Azo-TrEG@CD][PWV] solution (0.17 mg ml^−1^, 120 ml) and then grinding it into powder. The film sample was prepared by the filtration of [Azo-TrEG@CD][PWV] solution (0.18 mg ml^−1^, 120 ml) over a supporting filter (Whatman Nuclepore Track-Etched Polycarbonate Membrane; effective filtration area: 3.14 cm^2^; pore size: 200 nm), drying in oven at 40 °C for 48 h.

### Particle size calculation of CdTe QDs

The core diameter (*D*_c_) of CdTe QDs was calculated according to the following published equation^62^: *D*_c_=(9.8127 × 10^−7^)*λ*^3^−(1.7147 × 10^−3^)*λ*^2^+(1.0064)*λ*−194.84, where *λ* (nm) is the maximum wavelength corresponding to the first excitonic absorption peak of QDs. The full diameter of surface stabilized CdTe QDs (average diameter *D*) was calculated by summing the molecular length of ligands (*l*) and the calculated *D*_c_ value, where the molecular length of ligand stabilizer is estimated ∼0.46 nm for TG and ∼0.65 nm for MPA, simulated by ChemBio 3D (12.0 version). Thus, the diameters (*D*) of four QDs were calculated to be 3.3 nm for TG-1, 4.4 nm for TG-2, 4.0 nm for MPA-1 and 4.8 nm for MPA-2.

## Additional information

**How to cite this article:** Yue, L. *et al.* Flexible single-layer ionic organic-inorganic frameworks towards precise nano-size separation. *Nat. Commun.* 7:10742 doi: 10.1038/ncomms10742 (2016).

## Supplementary Material

Supplementary Information Supplementary Figures 1-52, Supplementary Tables 1-5 and Supplementary References

## Figures and Tables

**Table 1:** Summary of separation efficiency of Tr/Te-membranes for QDs solutions.

	QDs separation efficiency*	
	**Tr-membrane**	**Te-membrane**
TG-1	76.3%	93.4%
TG-1+TG-2	73.4%	81.3%

QDs, quantum dots.

^*^The separation efficiency was calculated from comparison of relative fluorescence intensity at emission wavelength 523 and 533 nm (for Tr and Te membranes, respectively) before and after the filtration.
