# Supplementary material for: Flexible single-layer ionic organic–inorganic frameworks towards precise nano-size separation
Source: Nat Commun. 2016 Feb 29;7:10742. doi: 10.1038/ncomms10742 (PMC4773479; doi:10.1038/ncomms10742)
Supplement: Supplementary Information — Supplementary Figures 1-52, Supplementary Tables 1-5 and Supplementary References [file ncomms10742-s1.pdf]

Supplementary Figures

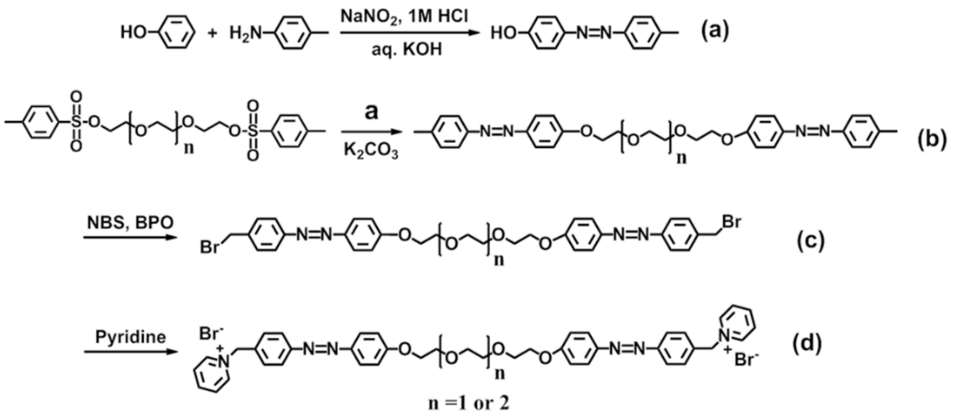

Supplementary Figure 1: Synthetic route. Azo-Tr/TeEG ·2Br (TrEG: n = 1; TeEG: n = 2).

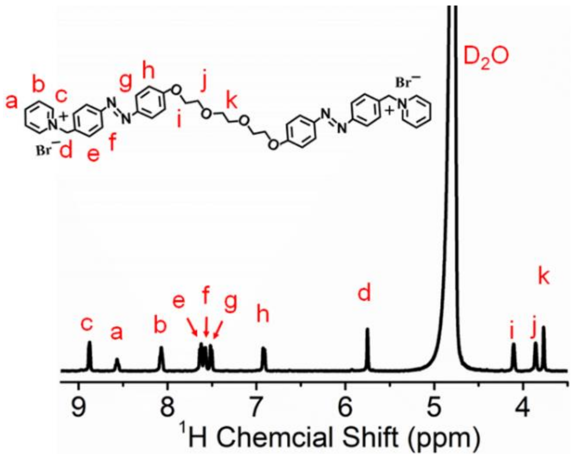

Supplementary Figure 2: ^{1}H NMR spectrum. Azo-TrEG ·2Br in D_2O at 25 ℃.

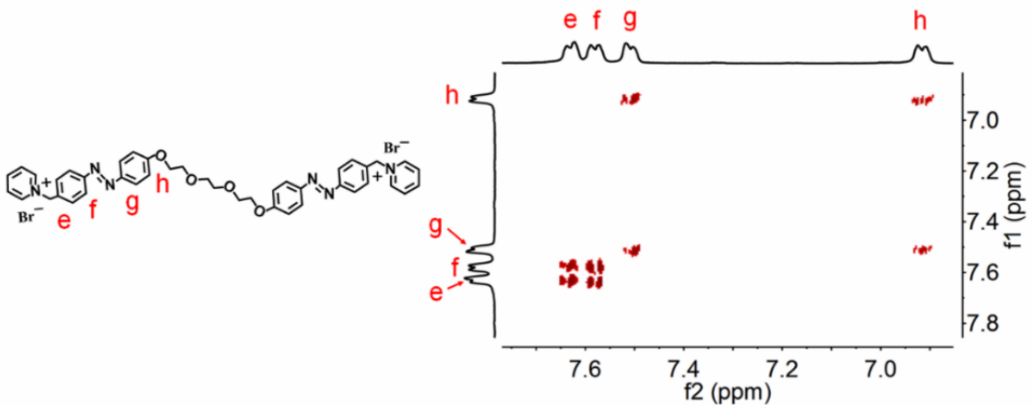

Supplementary Figure 3: 2D COSY NMR spectrum. Azo-TrEG ·2Br in D_2O at 25 ℃.

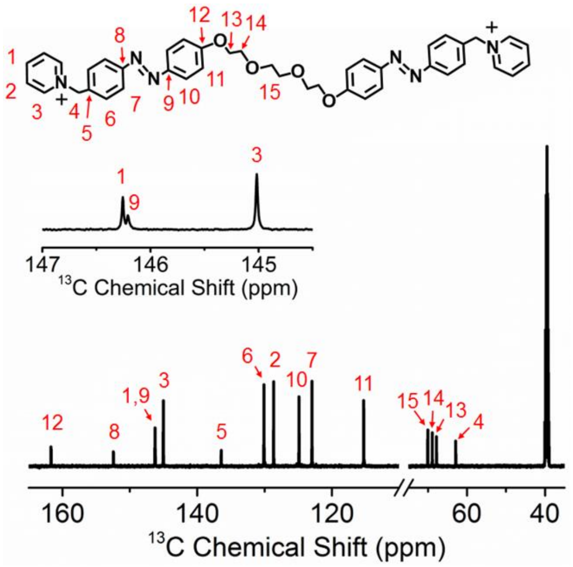

Supplementary Figure 4: ^{13}C NMR spectrum. Azo-TrEG·2Br in DMSO-d_6 at 25 °C.

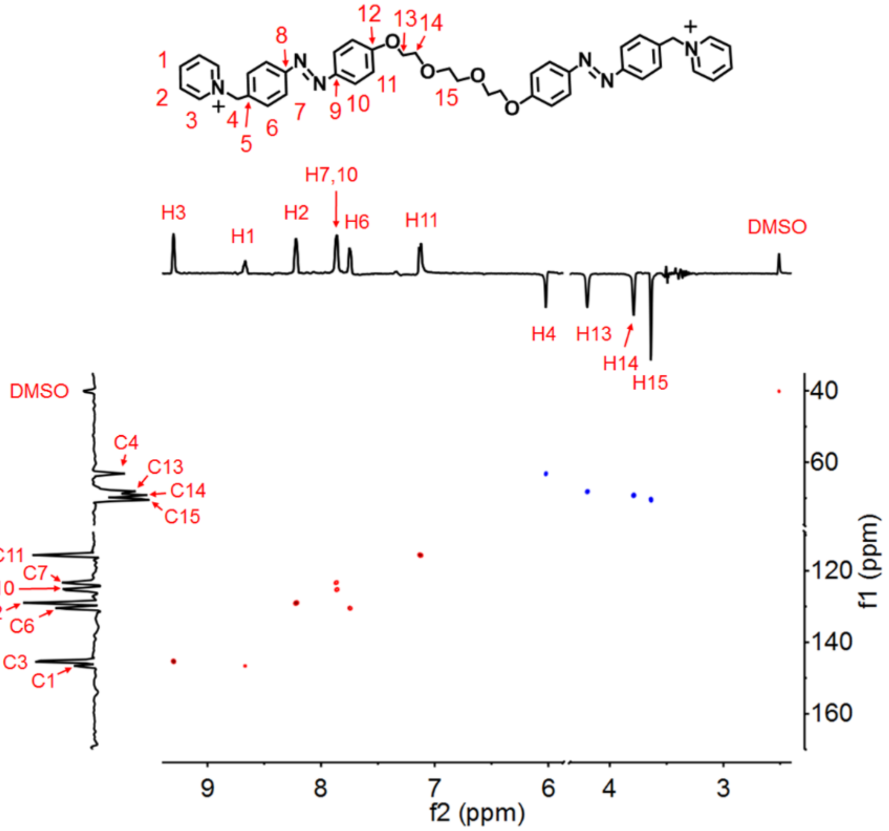

Supplementary Figure 5: 2D HSQC NMR spectrum. Azo-TrEG·2Br in DMSO-d_6 at 25 °C.

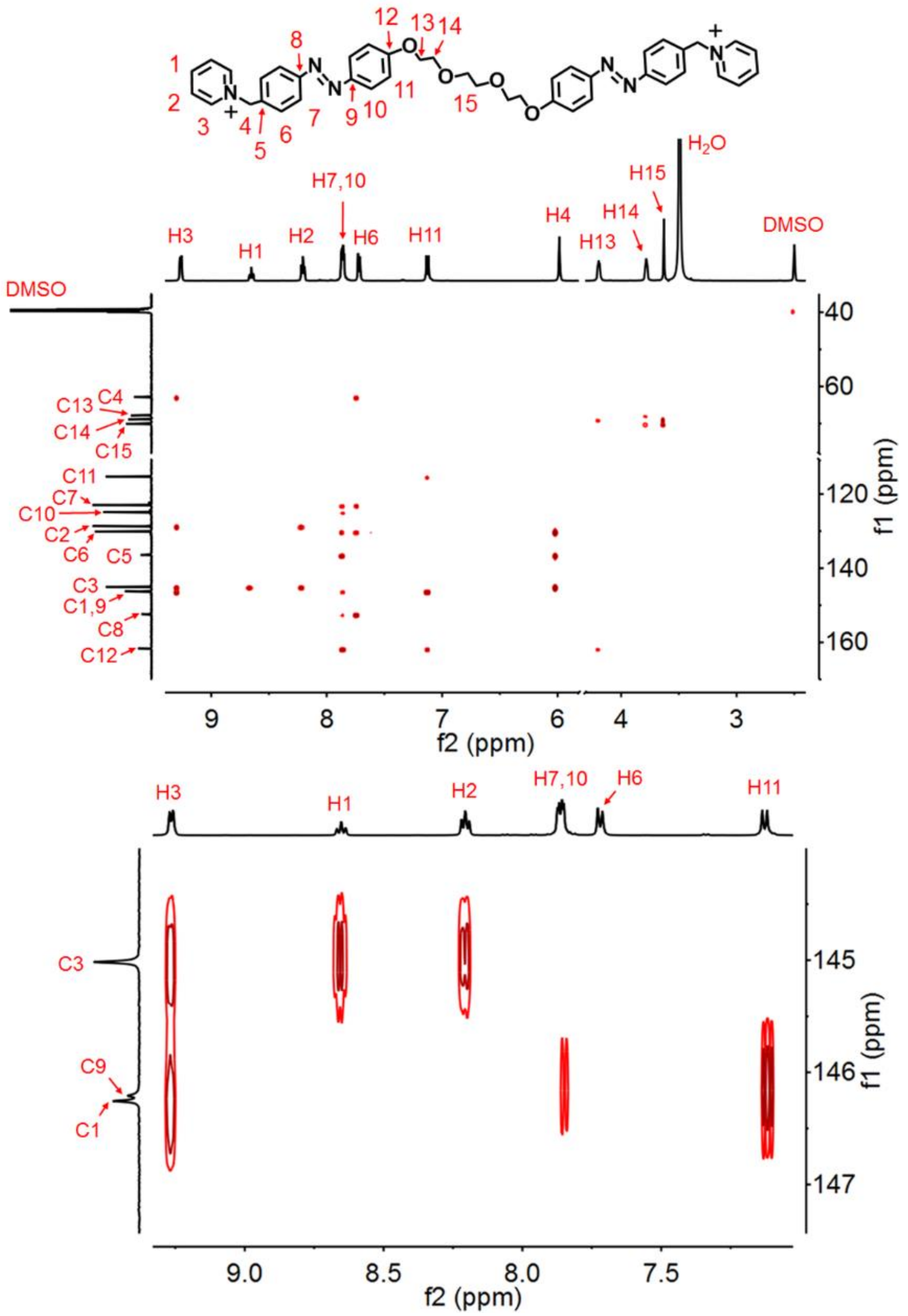

Supplementary Figure 6: 2D HMBC NMR spectrum. Azo-TrEG ·2Br in DMSO-d_6 at 25 ℃.

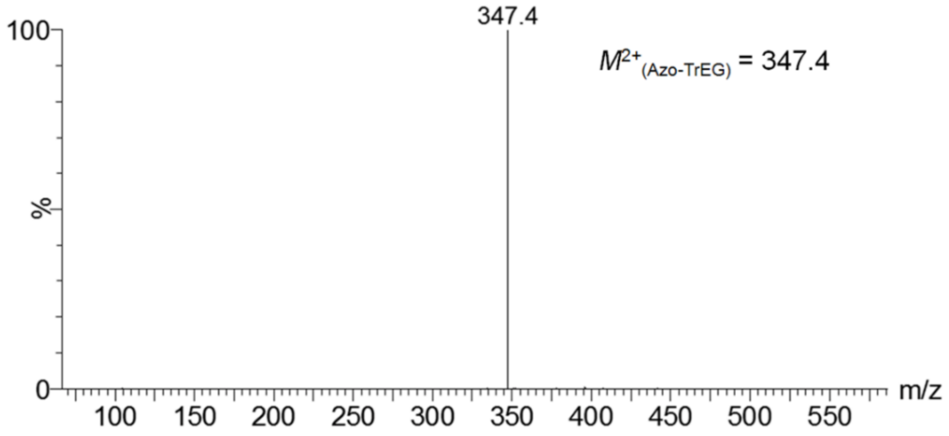

Supplementary Figure 7: ESI-MS. Azo-TrEG 2Br.

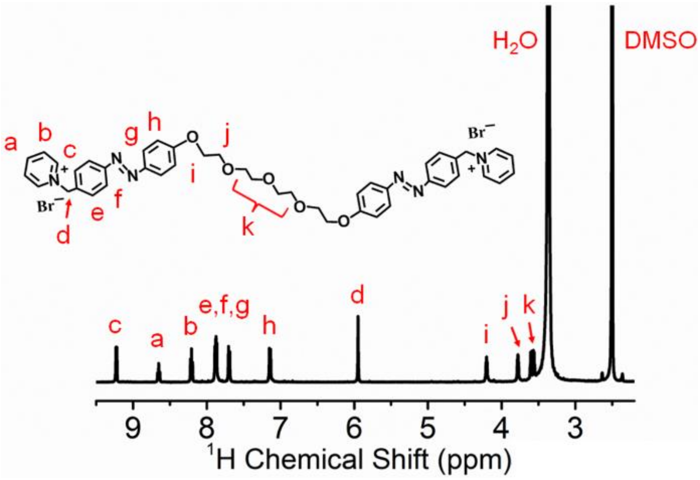

Supplementary Figure 8: ^{1}H NMR spectrum. Azo-TeEG 2Br in DMSO-d_6 at 25 ℃.

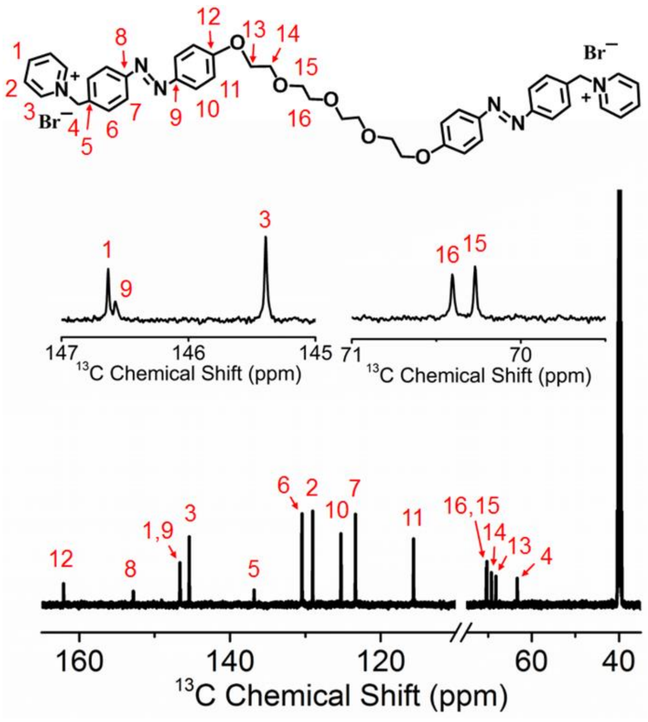

Supplementary Figure 9: ^{13}C NMR spectrum. Azo-TeEG ·2Br in DMSO-d_6 at 25 ℃.

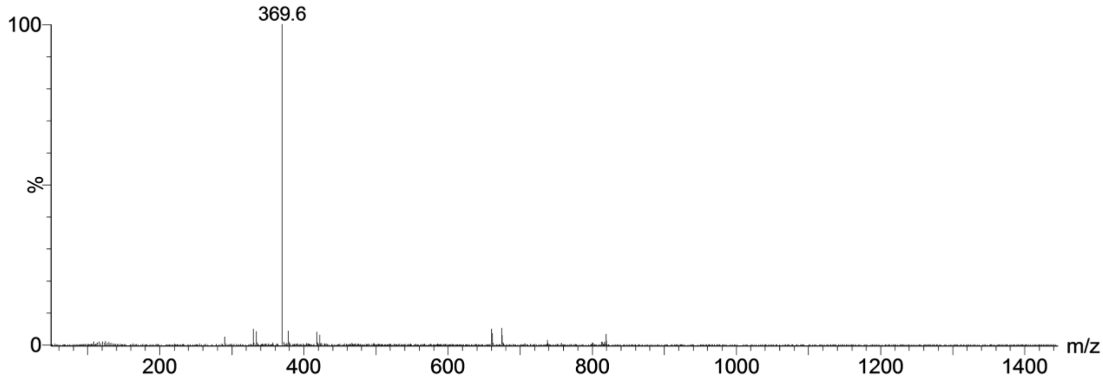

Supplementary Figure 10: ESI-MS. Azo-TeEG ·2Br. The mass peak at 369.6 belongs to $[Azo\text{-}TeEG]^{2+}$ (calcd. value: 369.2).

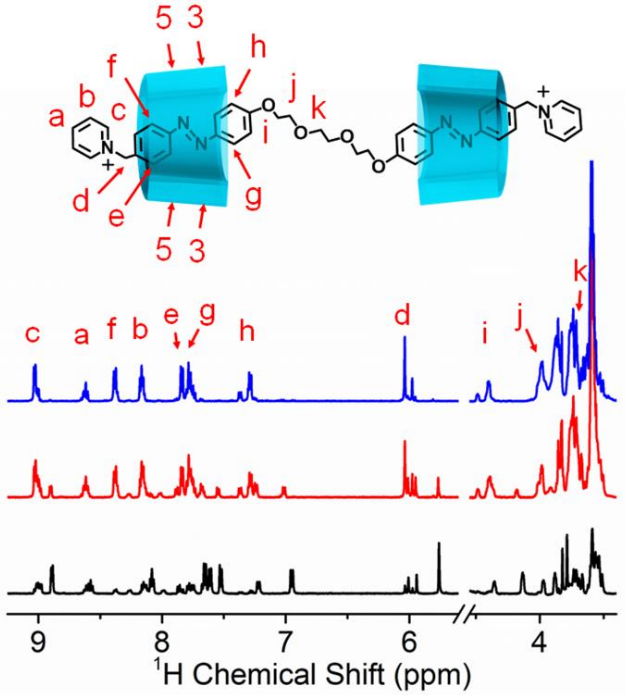

Supplementary Figure 11: ^{1}H NMR spectra. Azo-TrEG 2Br upon addition of 0.5 (black line), 1.5 (red line), 2.5 (blue line) eq. CDs in D_2O at 25 ℃.

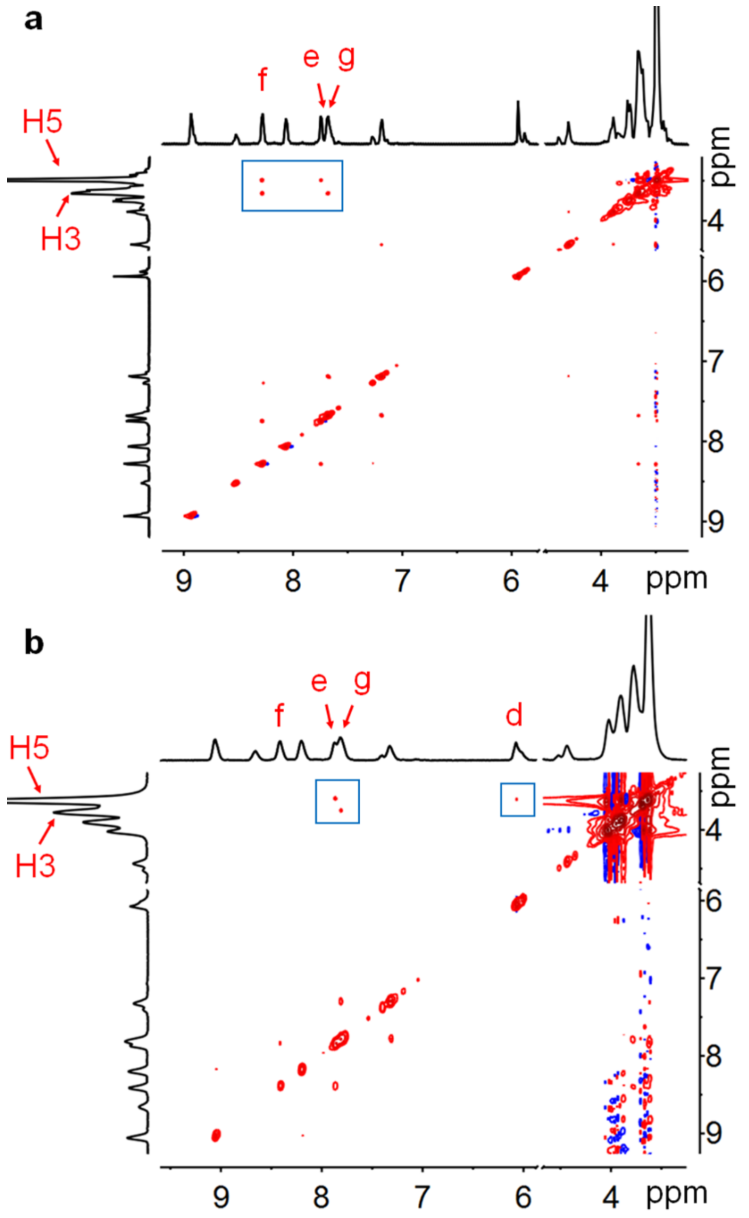

Supplementary Figure 12: 2D NOESY NMR spectra. (**a**) Azo-TrEG@CD·2Br, and (**b**) [Azo-TrEG@CD][PWV] in D_2O at 25 ℃.

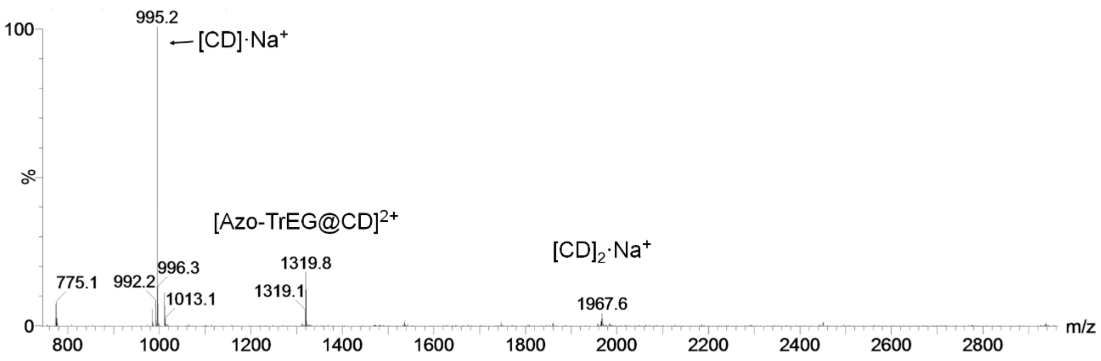

Supplementary Figure 13: ESI-MS. CD and Azo-TrEG ·2Br mixture in 5:1. The peak at 775.1 belongs to [Azo-TrEG ·Br]$^{+}$ (calcd. value: 774.7); the peak at 995.2 belongs to [CD ·Na]$^{+}$ (calcd. value: 995.3); the peak at 1319.8 belongs to [Azo-TrEG@CD]$^{2+}$ (calcd. value: 1320.2), and the peak at 1967.6 belongs to [CD$_2$ ·Na]$^{+}$ (calcd. value: 1967.6).

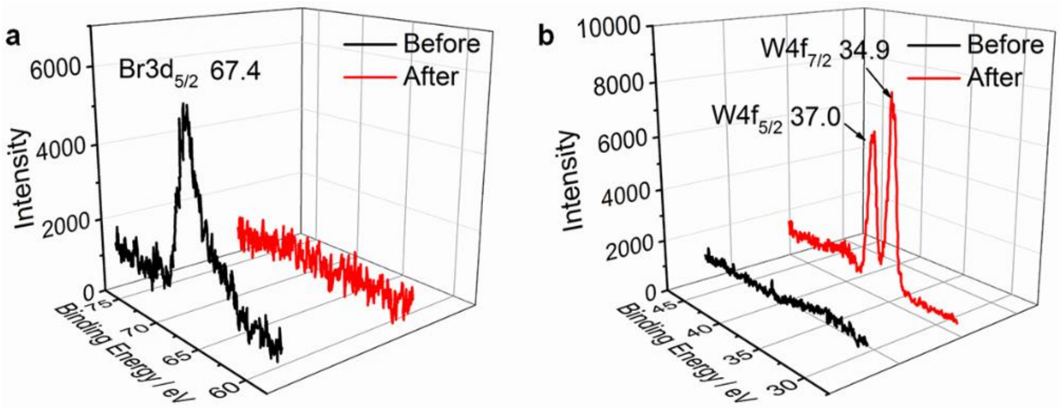

Supplementary Figure 14: XPS spectra. (**a**) Br (3d) and (**b**) W (4f) elements of Azo-TrEG@CD ·2Br inclusion complex before (black line) and after (red line) mixing with PWV^{4-} and then passing through a filtration.

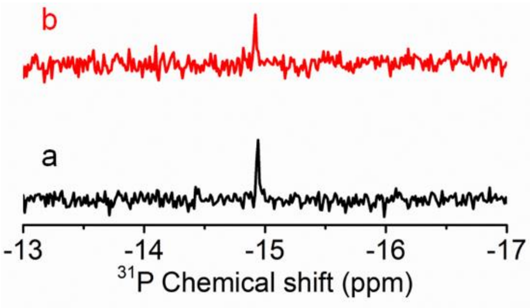

Supplementary Figure 15: ^{31}P NMR spectra. (**a**) PWV^{4-} cluster and (**b**) [Azo-TrEG@CD][PWV] in D$_2$O, 25 ℃.

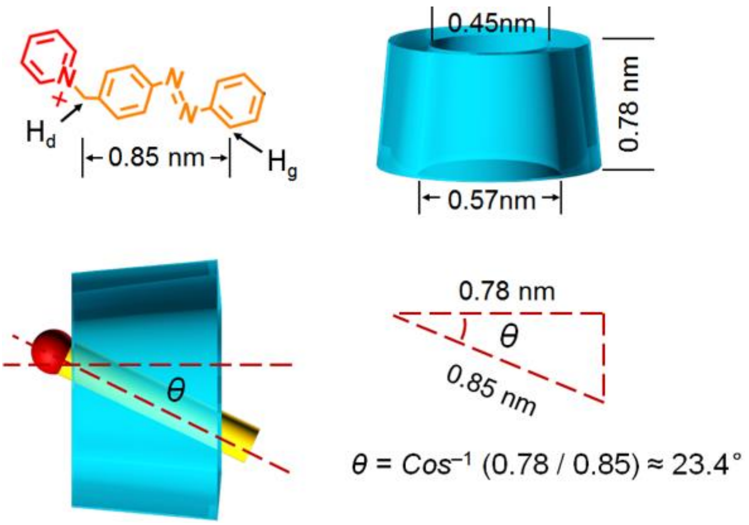

Supplementary Figure 16: Calculation on the orientation angle of Azo group included in CD cavity. According to the correlation signals in 2D NOESY NMR spectrum (Supplementary Fig. 12b), the protons from H(d) to H(g) are incorporated in the cavity. Based on the molecular length from H(d) to H(g) (0.85 nm) and the height of CD's cavity (0.78 nm), the calculated angle (θ) between long axis of Azo group and the normal of CD is around 23.4 °.

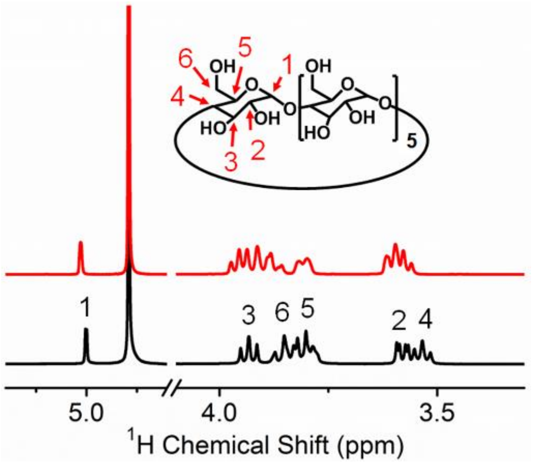

Supplementary Figure 17: ^{1}H NMR spectra. CD without (black line) and with (red line) addition of 0.25 eq. PWV^{4-} in D_2O at 25 ℃. The largest downfield shift (about 0.06 ppm) for proton H(6) is observed, implying the interaction of PWV^{4-} with the narrow ring of CD.

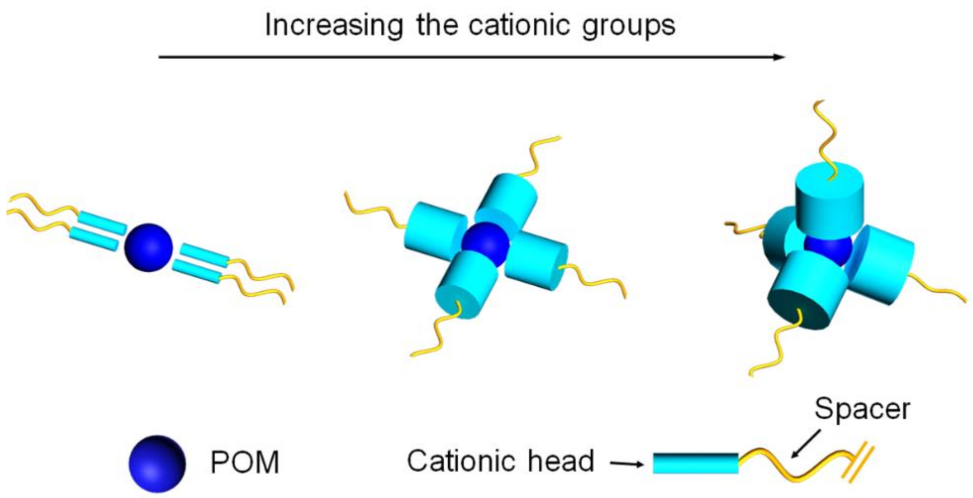

Supplementary Figure 18: Ionic self-assembly models of anionic POM and cationic groups with size gradually increased. Due to the dynamic and non-directed binding features of ionic interactions, the fluid negative charges of POMs[S1,S2] allow even distribution of cationic head groups upon charge neutralization. However, driven by additional interactions occurring between cationic organic groups, such as hydrophilic/hydrophobic interactions[S3–S7], uneven distribution becomes reasonable. Taking four-charge POM covered by four cationic units as an example, there are several possibilities for four cations' packing around the POM, depending on their size match relationship and interactions between cationic parts. When the POM's surface area is not fully occupied, the symmetric distribution of counterions directing to lamellar self-assembled structure (left), driven by the lateral interaction or the requirement for tight packing, would be dominated. Increasing the size of cationic head group, more surface area of the POM will be occupied, and the planar distribution becomes favorable (middle). While further increasing the size of cationic head, the POM's surface area could not provide enough space for a planar packing and a three dimensional distribution of the counterions will become main covering style (right). In general, the packing form of cations around a POM is in close relation to the geometrical relationship of each component and the additional interactions.

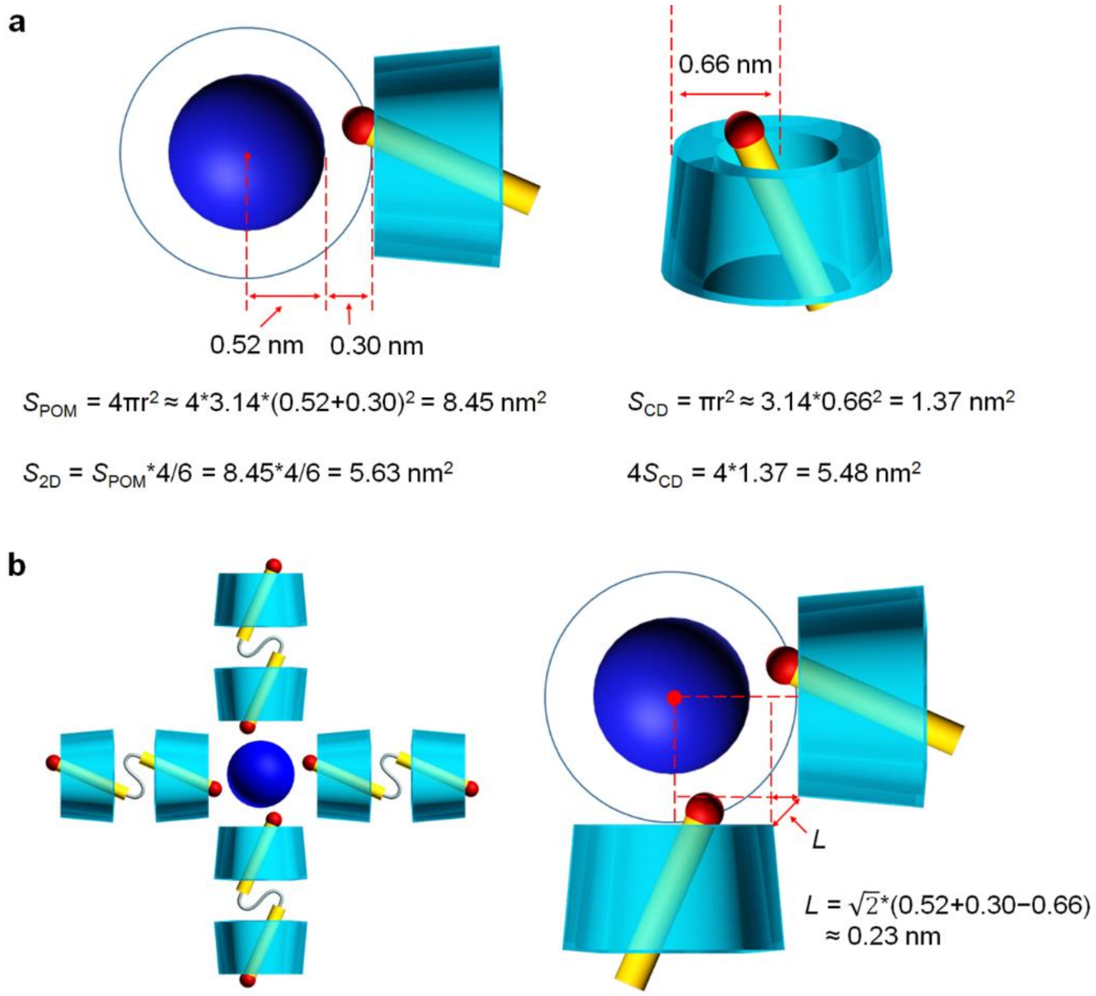

Supplementary Figure 19: Structural matching relation of four CD-shielded cations Azo-TrEG@CD^{2+} surrounding one anionic PWV^{4-}. For a simple comprehension, the POM cluster can be divided into six identical regions similar to the faces of a cube. (**a**) Calculation for structure and matching: the surface area of POM (S_{POM}) is 8.45 nm^2, the surface area of POM in a 2D plane (S_{2D}) is 5.63 nm^2, the area of single CD-shielded cationic head (S_{CD}) is 1.37 nm^2, and the total area of four cationic heads (4S_{CD}) around one POM is 5.48 nm^2, very close to S_{2D}; (**b**) The schematic drawing of proposed self-assembly model: 2D crossing planar arrangement (left), the distance (*L*) between narrow rings of neighboring CDs is estimated ca. 0.23 nm (right). Here, 0.52 nm is the radius of PWV^{4-} (from crystal structure), 0.30 nm is the distance between PWV^{4-} and CD shield (from general electrostatic interaction distance and consideration of the cationic head's location), and 0.66 nm is the radius of the narrow ring of CD (from crystal structure).

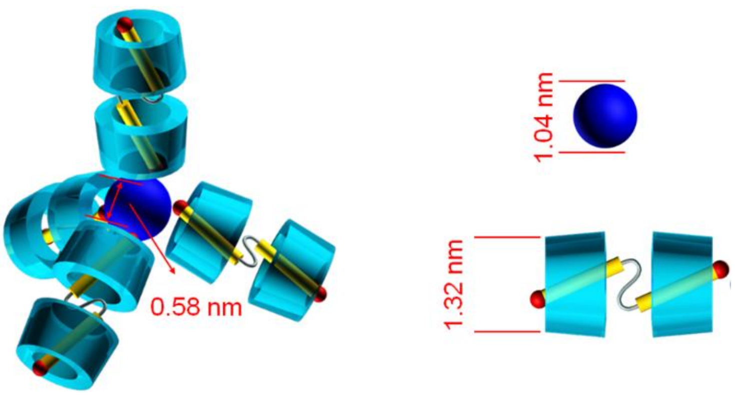

Supplementary Figure 20: The tetrahedron structure model based on PWV^{4-} and Azo-TrEG@CD^{2+}. The estimated distance between narrow rings of neighboring CDs (*L*) is ca. 0.58 nm, calculated by using the structural information given in Supplementary Fig. 19.

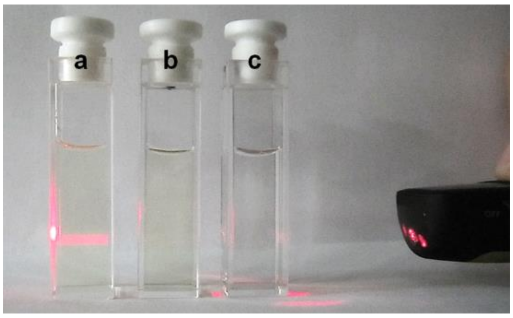

Supplementary Figure 21: Digital photographs. (**a**) [Azo-TrEG@CD][PWV], (**b**) Azo-TrEG@CD·2Br and (**c**) PWV^{4-} aqueous solution.

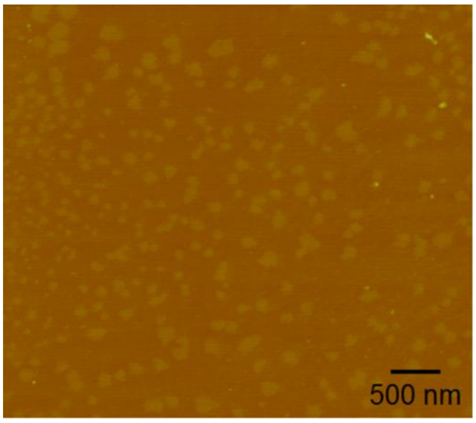

Supplementary Figure 22: AFM image. [Azo-TrEG@CD][PWV] self-assembly after aging for 3 min.

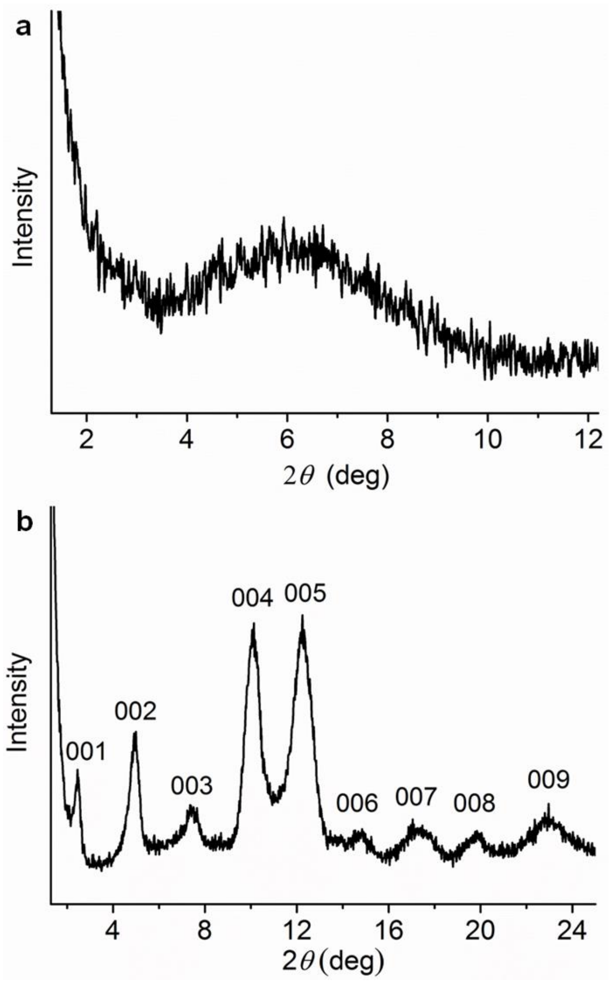

Supplementary Figure 23: XRD patterns. (**a**) The powdered sample prepared by solution freeze-drying and (**b**) the film sample prepared by solution filtration.

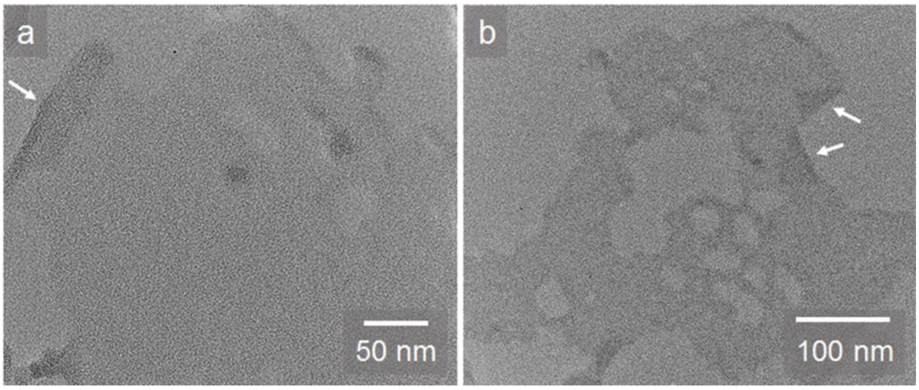

Supplementary Figure 24: TEM images. [Azo-TrEG@CD][PWV] self-assembly. Wrinkle structure in (**a**) and folding structure in (**b**) are observed at the edge of single-layer sheet (pointed by white arrows).

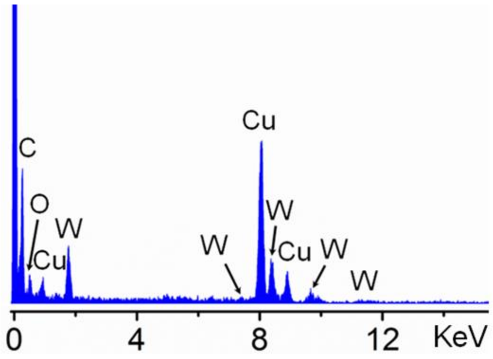

Supplementary Figure 25: EDX spectrum. [Azo-TrEG@CD][PWV] self-assembly.

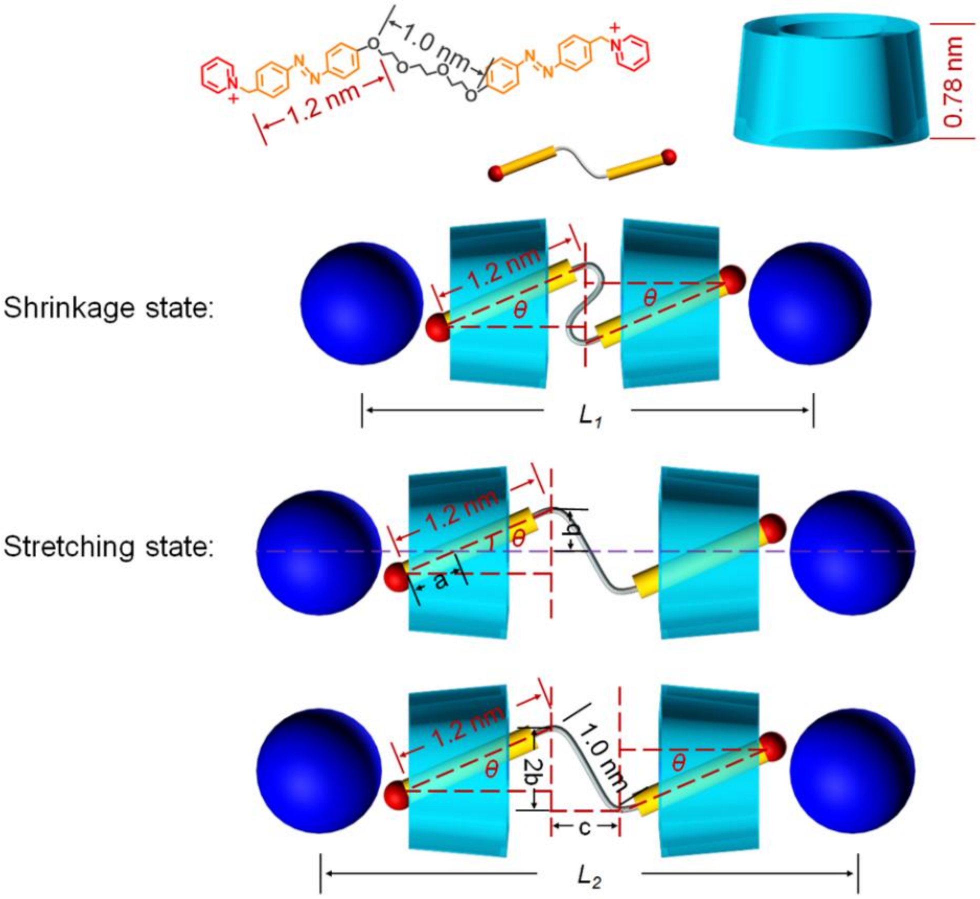

Supplementary Figure 26: Calculation for distance between two PWV^{4-} clusters connected by Azo-TrEG@CD^{2+}. For shrinkage state, the distance $L_1 = (1.2 \times \cos\theta + 0.52 + 0.30) \times 2 \approx 3.8$ nm. For the stretching state, a purple dotted line is set as the centerline along the whole Azo-TrEG@CD^{2+} and PWV^{4-} cluster structure, where *a* denotes half of the length of azobenzene guest group included in CD (0.85/2 ≈ 0.43 nm), the height $b = (1.2 - a) \times \sin\theta \approx 0.31$ nm and the length $c = \sqrt{1.0^2 - 0.62^2} \approx 0.78$ nm, and thus the distance $L_2 = (1.2 \times \cos\theta + 0.52 + 0.30) \times 2 + 0.78 \approx 4.6$ nm. Where θ value is ca. 23.4 °(Supplementary Fig. 16), 0.30 nm is the distance between PWV^{4-} and CD shield, and 0.52 nm is the radius of PWV^{4-}.

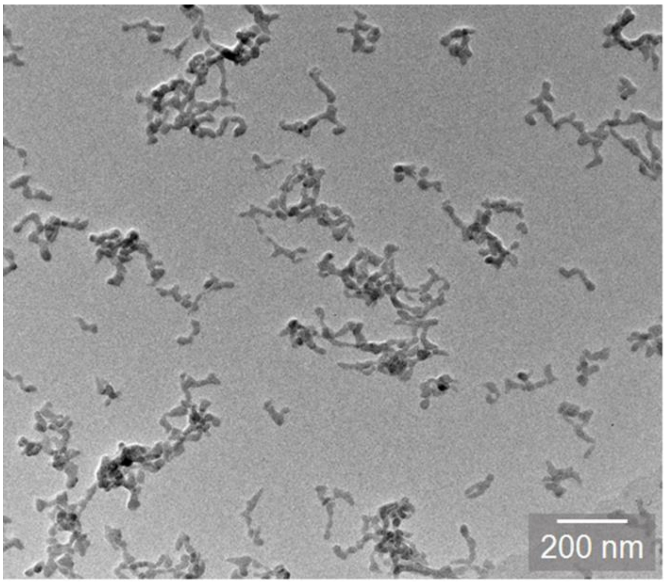

Supplementary Figure 27: TEM image. Self-assembly of Azo-TrEG@**β-CD**$^{2+}$ and PWV^{4-} mixture at molar ratio 2:1.

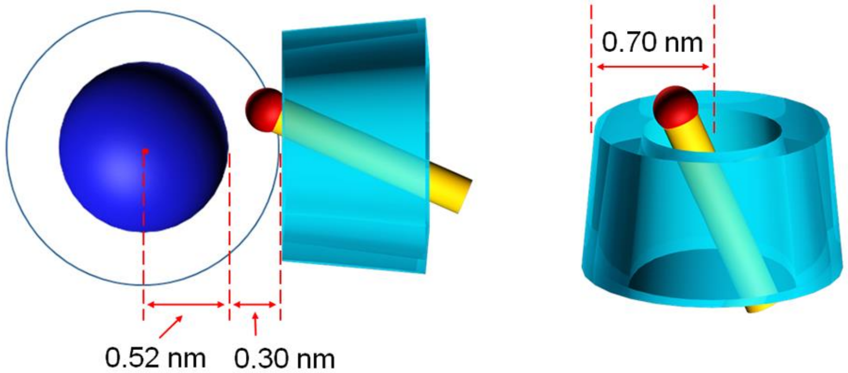

$$S_{POM} = 4\pi r^2 \approx 4*3.14*(0.52+0.30)^2 = 8.45\ nm^2$$

$$S_{\beta\text{-}CD} = \pi r^2 \approx 3.14*0.70^2 = 1.54\ nm^2$$

$$S_{2D} = S_{POM}*4/6 = 8.45*4/6 = 5.63\ nm^2$$

$$4S_{\beta\text{-}CD} = 4*1.54 = 6.15\ nm^2$$

$$S_{2D} < 4S_{\beta\text{-}CD}$$

Supplementary Figure 28: Structure relation of four Azo-TrEG@β-CD^{2+} surrounding one PWV^{4-} cluster. For understanding conveniently, POM cluster could be divided into six identical regions like a cube. Based on the structural information of all building components, the surface area of POM (S_{POM}) is 8.45 nm^2; the surface area of POM in 2D plane (S_{2D}) is 5.63 nm^2, the lateral area of single β-CD-shielded cationic head (S_{CD}) is 1.54 nm^2, and the total area of four cationic heads ($4S_{CD}$) around the POM becomes 6.15 nm^2. For more information, the radius of PWV^{4-} is 0.52 nm, the distance between PWV^{4-} and β-CD shield is 0.30 nm, and the radius of the narrow ring of β-CD is 0.70 nm.

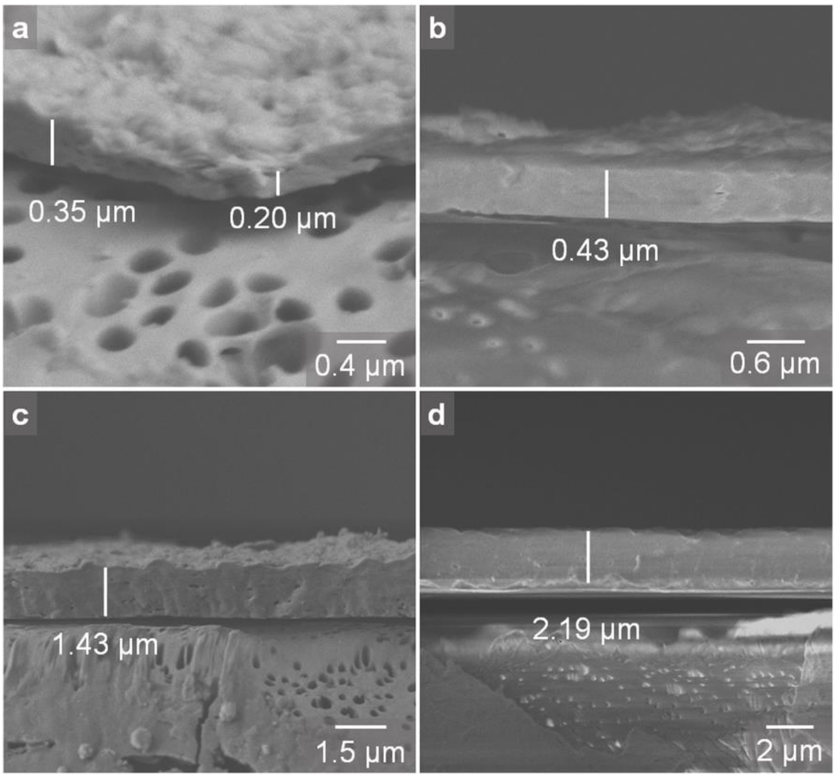

Supplementary Figure 29: Cross-sectional SEM images. [Azo-TrEG@CD][PWV] membranes prepared by filtration of 20 mL sample solutions with the concentration of: (**a**) 0.02, (**b**) 0.04, (**c**) 0.06, and (**d**) 0.08 mg mL^{-1}.

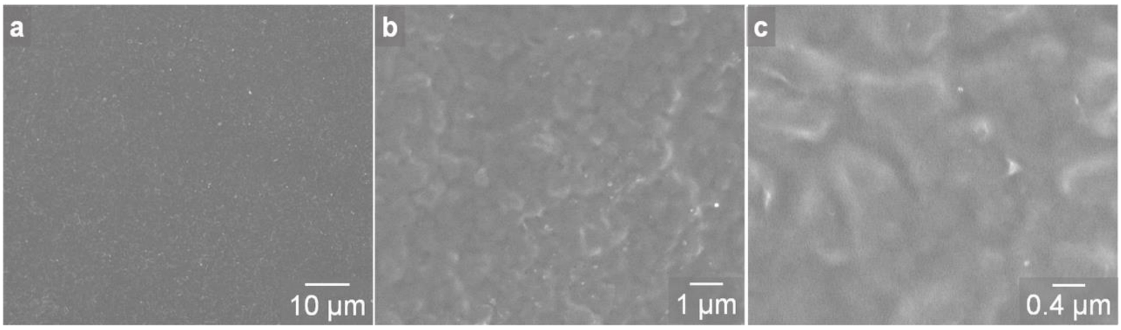

Supplementary Figure 30: SEM images. Isolated [Azo-TrEG@CD][PWV] membrane in scale: (**a**) large, (**b**) middle, and (**c**) small.

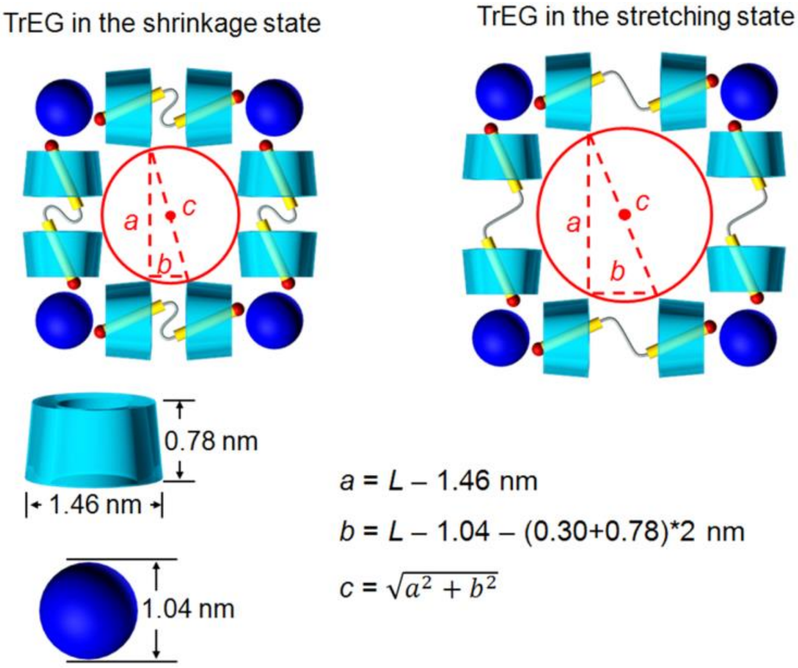

Supplementary Figure 31: Mesh scale calculation for ionic organic-inorganic framework (IOIF) self-assembly of Azo-TrEG@CD^{2+} with PWV^{4-}. Because *L* is estimated between 3.8 and 4.6 nm based on TrEG chains' shrinkage and stretching, the mesh diameter *c* is calculated between 2.4 and 3.4 nm, Where 0.30 nm represents the distance between the surface of CD's narrow ring and anionic PWV^{4-}, 1.04 nm comes from the diameter of PWV^{4-}, 1.46 nm and 0.78 nm point the broad ring diameter and the height of α-CD.

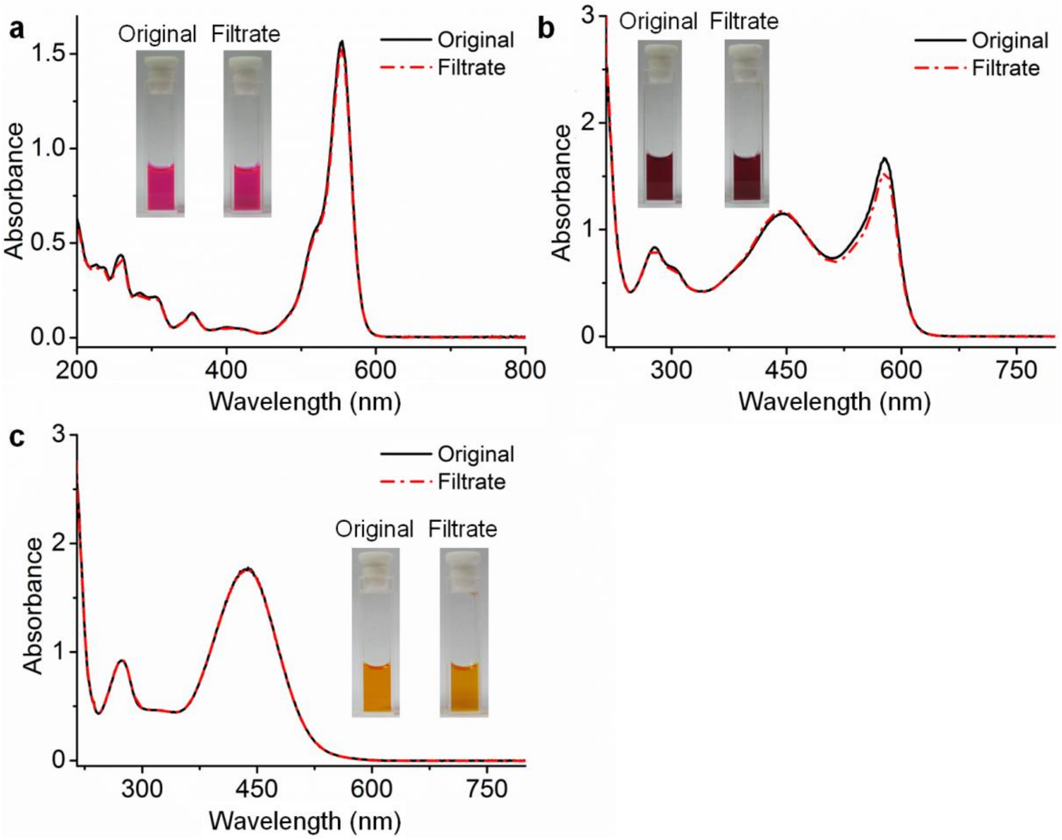

Supplementary Figure 32: UV-Vis spectra and corresponding photographs. (**a**) Rhodamine B, (**b**) xylenol orange at pH = 7.9, and (**c**) xylenol orange at pH = 4.0, with and without filtration through Tr-membrane. All sample solutions were diluted for spectral measurement.

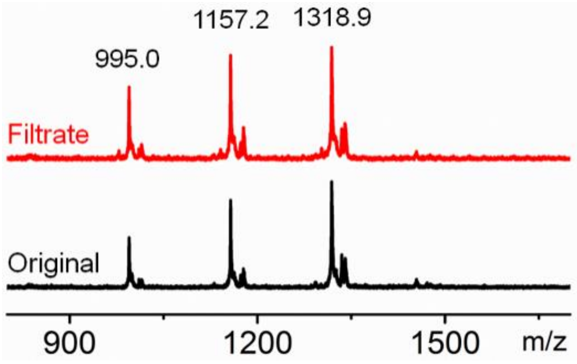

Supplementary Figure 33: MALDI-TOF MS. Initial mixture of α-CD, β-CD and γ-CD and the filtrate passing through Tr-membrane. The peak at 995.0 belongs to [α-CD·Na]$^+$ (calcd. value: 995.3), while the peak at 1157.2 and 1318.9 denote to [β-CD·Na]$^+$ (calcd. value: 1157.4), and [γ-CD·Na]$^+$ (calcd. value: 1319.4).

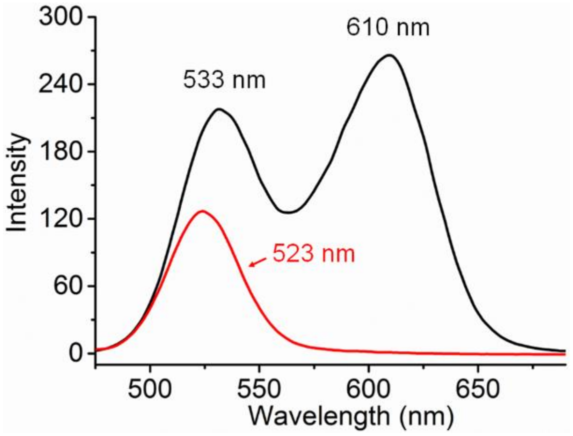

Supplementary Figure 34: Fluorescent spectra. The QDs mixture solution of TG-1 (λ_{max} = 533 nm, D = 3.3 nm) and TG-2 (λ_{max} = 611 nm, D = 4.4 nm) (black line) and its filtrate (λ_{max} = 523 nm, D = 3.0 nm) screened by using Tr-membrane dried in the oven at 40 ℃ for 48 h (red line).

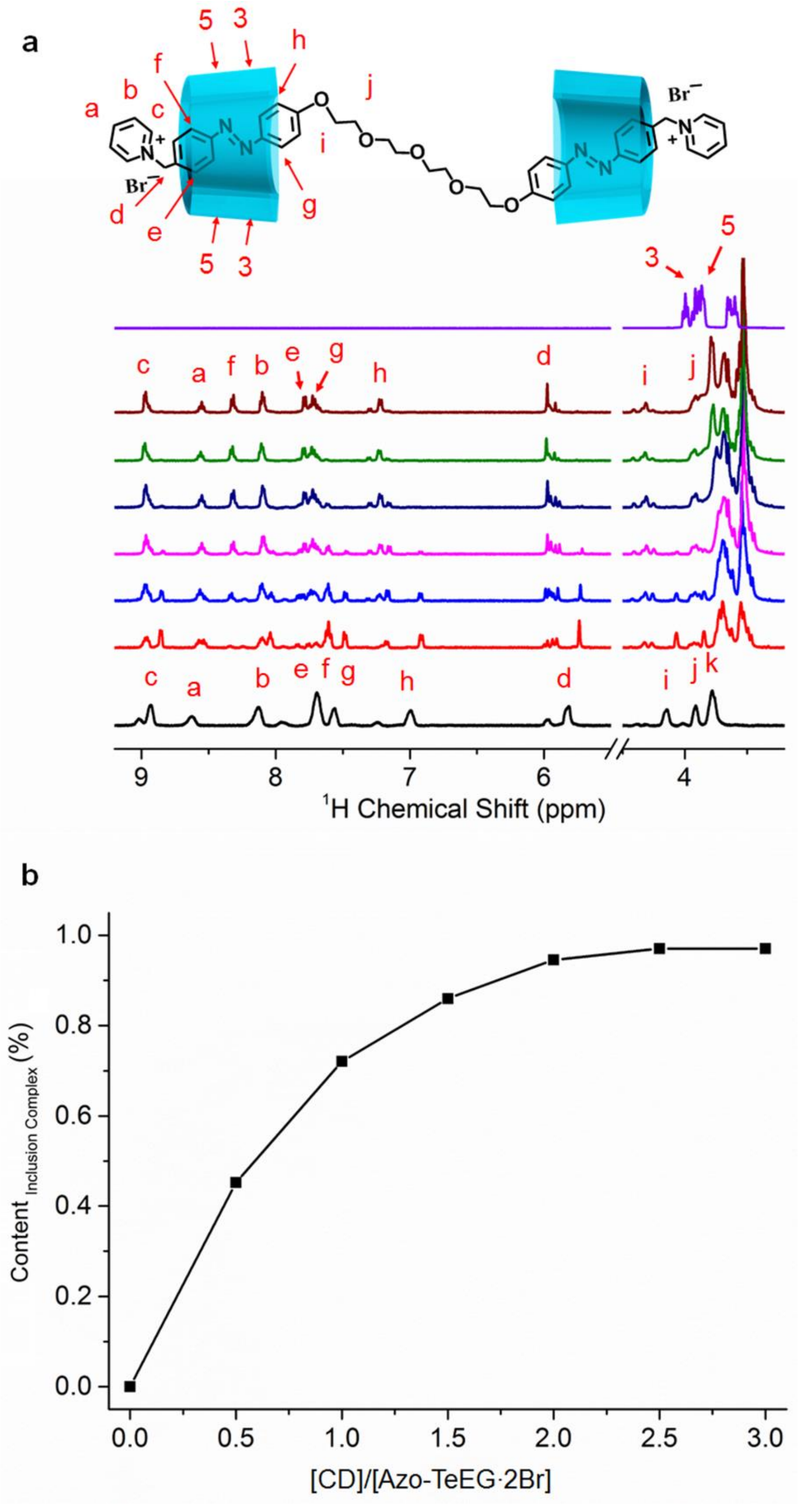

Supplementary Figure 35: ^{1}H NMR spectra. (**a**) Chemical shift of Azo-TeEG·2Br upon addition of 0 (black line), 0.5 (red line), 1.0 (blue line), 1.5 (magenta line), 2.0 (navy line), 2.5 (olive line), and 3.0 (wine line) eq. CDs, and pure CDs (violet line) in D_2O at 25 ℃; (**b**) the plot of relative content of inclusion complexes calculated from integral area of H(d) versus the molar ratio of CD to Azo-TeEG·2Br. Over 94% Azo-TeEG·2Br exists in pseudorotaxane state in 2:1 stoichiometry (Azo-TeEG@CD·2Br).

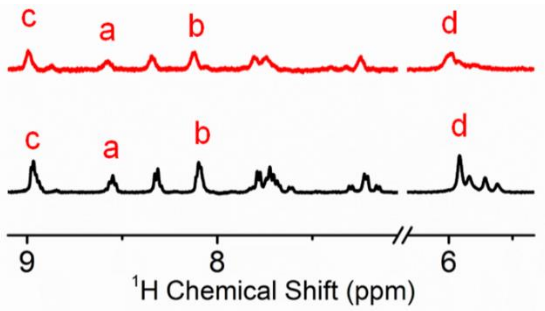

Supplementary Figure 36: ^{1}H NMR spectra. Azo-TeEG@CD·2Br in D_2O at 25 ℃ upon addition of 0 (black line) and 0.5 (red line) eq. PWV^{4-}. The signals of pyridinium head group H(a), H(b) and H(c) and the connecting methylene group H(d) shown in Supplementary Fig. 35 become wide and shift downfield, for instance 0.04 ppm for H(d), implying the existence of electrostatic interaction between Azo-TeEG@CD^{2+} and PWV^{4-}.

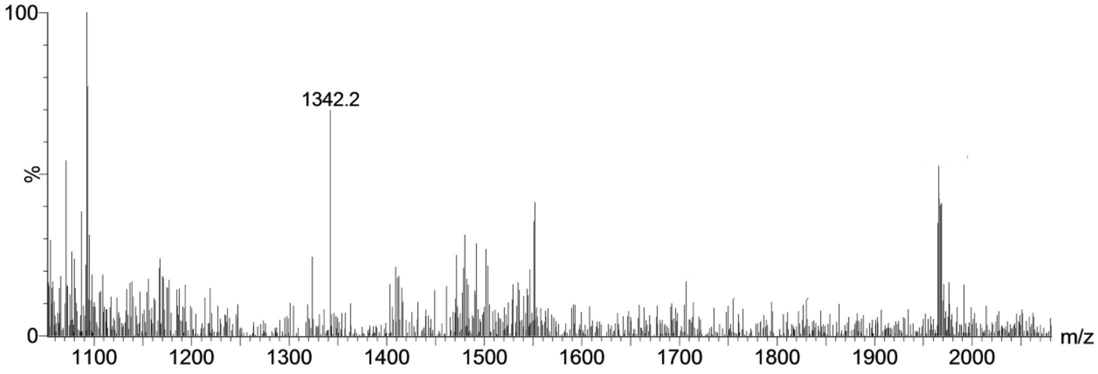

Supplementary Figure 37: ESI-MS. Azo-TeEG@CD·2Br, where the mass at 1342.2 is attributed to $[\text{Azo-TeEG@CD}]^{2+}$ (calcd. value: 1342.2).

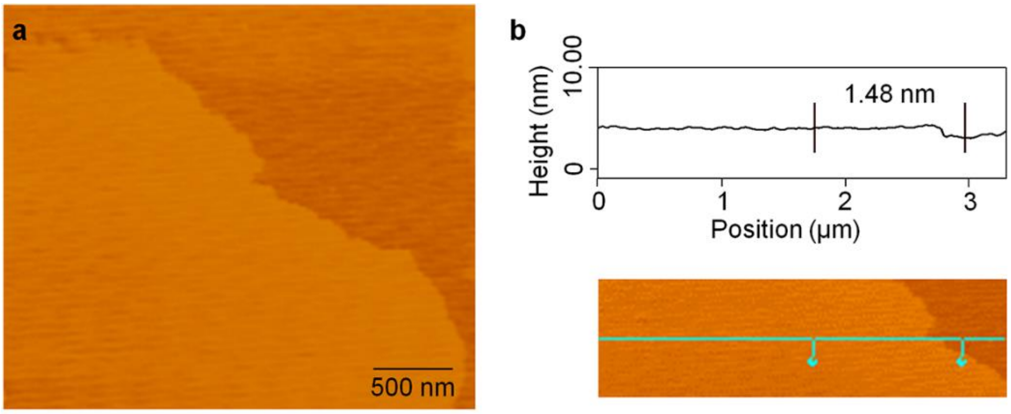

Supplementary Figure 38: AFM image. (**a**) Tapping-mode image and (**b**) height profile analysis of [Azo-TeEG@CD][PWV] self-assembly spreading on mica.

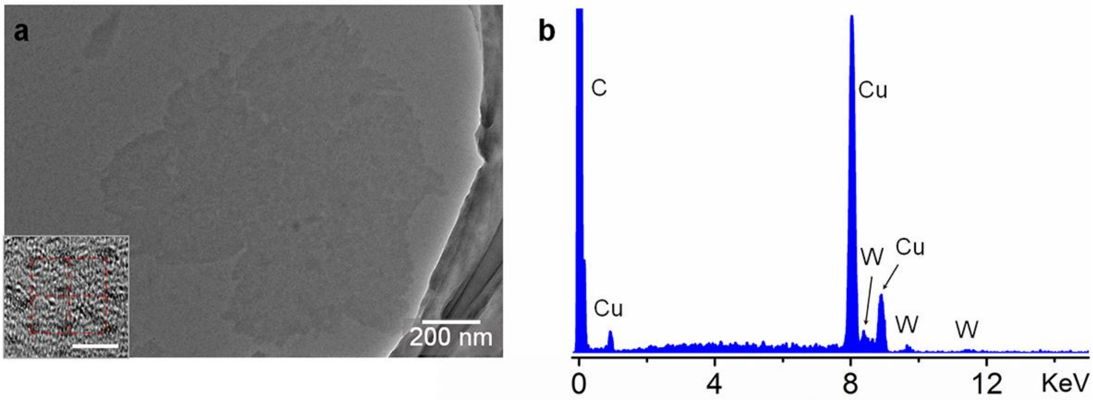

Supplementary Figure 39: TEM image and EDX spectrum. (**a**) TEM image of single-layer [Azo-TeEG@CD][PWV] self-assembly and (**b**) corresponding EDX spectrum. The scale bar in inset of **a** is 4 nm.

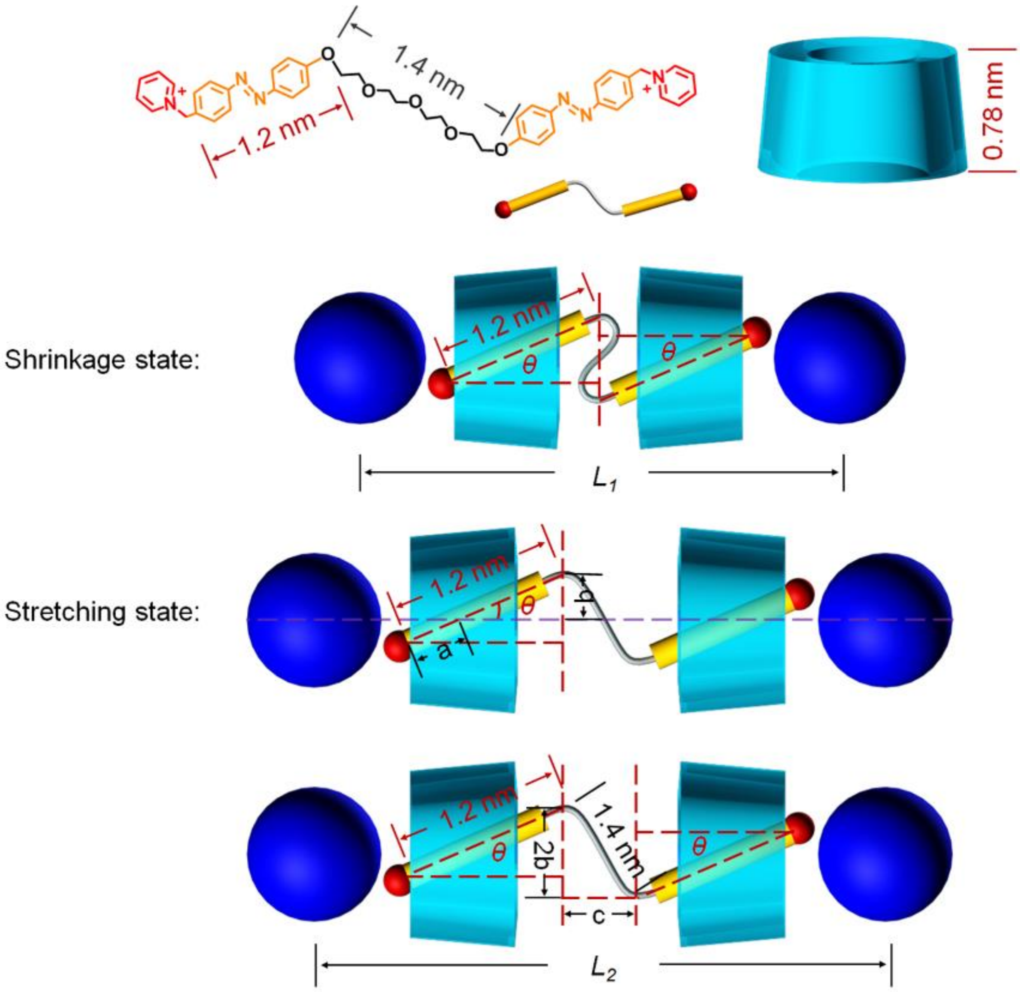

Supplementary Figure 40: Calculation of distance between PWV^{4-} clusters connecting by Azo-TeEG@CD^{2+}. The model is the same as that in Supplementary Fig. 26 with the changed length for TeEG (ca. 1.4 nm). The ideal lengths of planar square framework for L_1 and L_2 are 3.8 and 5.1 nm after considering the possible shrinkage and stretching of TeEG chain.

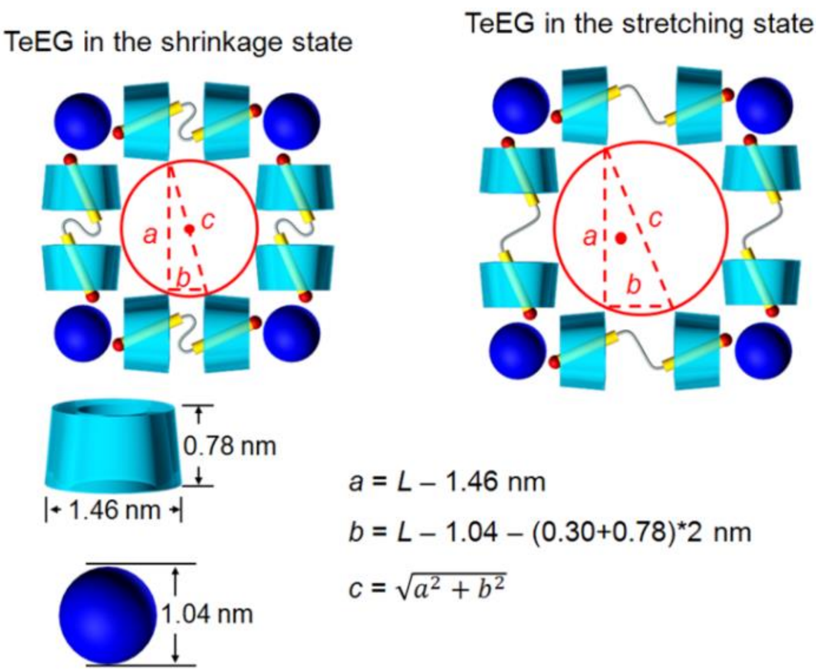

Supplementary Figure 41: Calculation for mesh diameter of prepared IOIF membrane comprised of Azo-TeEG@CD^{2+} with PWV^{4-}. The calculation model follows the same model as that shown in Supplementary Fig. 31. The mesh diameter *c* is estimated between 2.4 and 4.1 nm, based on the data from Supplementary Fig. 40 after considering the possible shrinkage and stretching of TeEG chain.

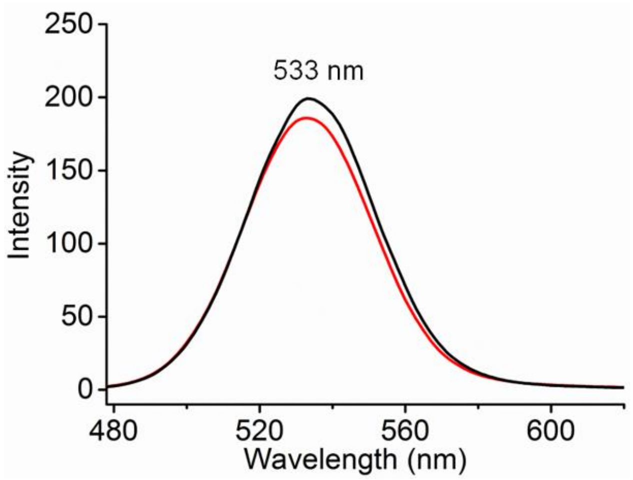

Supplementary Figure 42: Fluorescent spectra. Original solution of TG-1 QDs (λ_{max} = 533 nm, D = 3.3 nm, black line) and its filtrate (λ_{max} = 533 nm, D = 3.3 nm, red line) passing through Te-membrane.

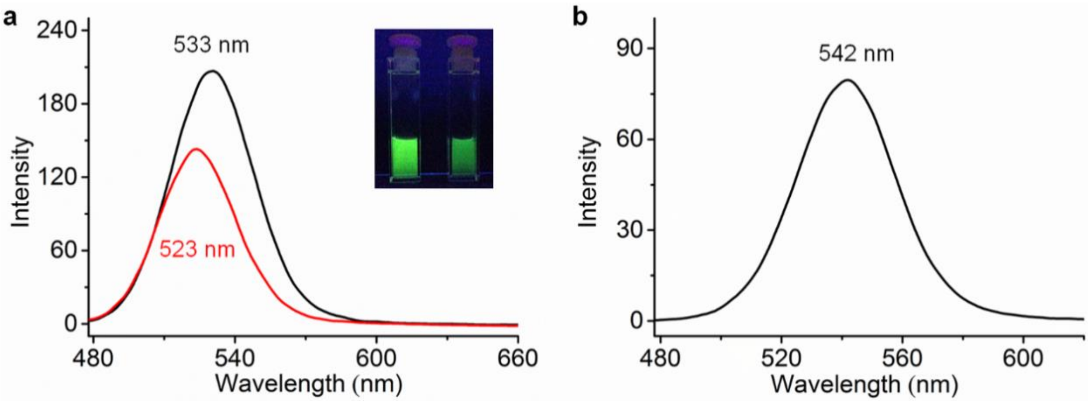

Supplementary Figure 43: Fluorescent spectra. (**a**) Original solution of TG-1 QDs (λ_{max} = 533 nm, D = 3.3 nm, black line, left cell) and the filtrate (λ_{max} = 523 nm, D = 3.0 nm, red line, right cell) screened by Tr-membrane, where the inset corresponding to photographs of samples before (left) and after filtration (right) under 365-nm light irradiation; (**b**) the residual QDs washed out from the filtrated membrane used in **a**.

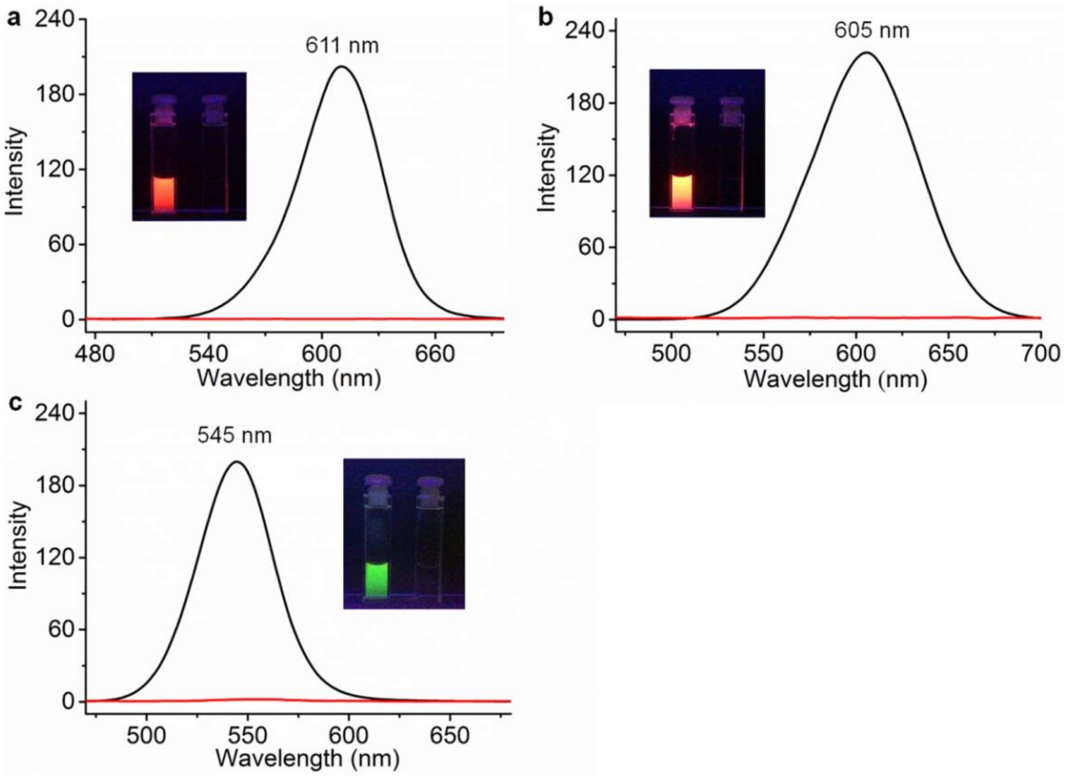

Supplementary Figure 44: Fluorescent spectra and corresponding photographs. (**a**) Original solution of TG-2 QDs (λ_{max} = 611 nm, D = 4.4 nm, black line, left cell) and the filtrate passed through Tr-membrane (red line, right cell); (**b**) original solution of MPA-2 QDs (λ_{max} = 606 nm, D = 4.8 nm, black line, left cell) and the filtrate treated by Tr-membrane (red line, right cell); and (**c**) original solution of MPA-1 QDs (λ_{max} = 545 nm, D = 4.0 nm, black line, left cell) and the filtrate screened by using Tr-membrane (red line, right cell). All photographs are taken under 365-nm light irradiation.

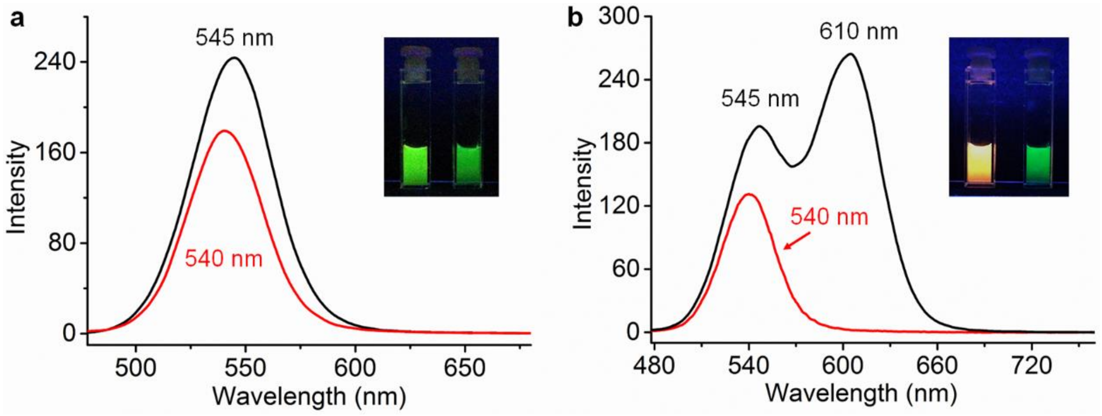

Supplementary Figure 45: Fluorescent spectra and corresponding photographs. (**a**) Original solution of MPA-1 QDs (λ_{max} = 545 nm, D = 4.0 nm, black line, left cell) and the filtrate treated by using Te-membrane (λ_{max} = 540 nm, D = 3.9 nm, red line, right cell); (**b**) original mixture solution of MPA-1 (λ_{max} = 545 nm, D = 4.0 nm) and TG-2 QDs (λ_{max} = 611 nm, D = 4.4 nm) (black line, left cell) and the filtrate passing through Te-membrane (λ_{max} = 540 nm, D = 3.9 nm, red line, right cell). The photographs in insets are taken under 365-nm light irradiation.

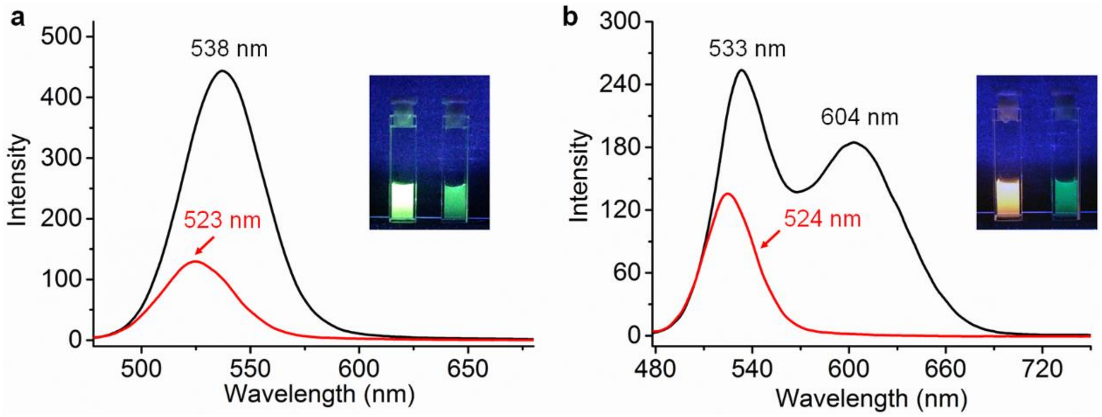

Supplementary Figure 46: Fluorescent spectra and corresponding photographs. (**a**) Original solution of MPA-1 (λ_{max} = 545 nm, D = 4.0 nm) and TG-1 (λ_{max} = 533 nm, D = 3.3 nm) (black line, left cell) QDs mixture and the filtrate screened by Tr-membrane (λ_{max} = 523 nm, D = 3.0 nm, red line, right cell); (**b**) original solution of TG-1 and MPA-2 (λ_{max} = 606 nm, D = 4.8 nm) (black line, left cell) QDs mixture and the filtrate screened with Tr-membrane (λ_{max} = 524 nm, D = 3.0 nm, red line, right cell). The inset photographs are taken under 365-nm light irradiation.

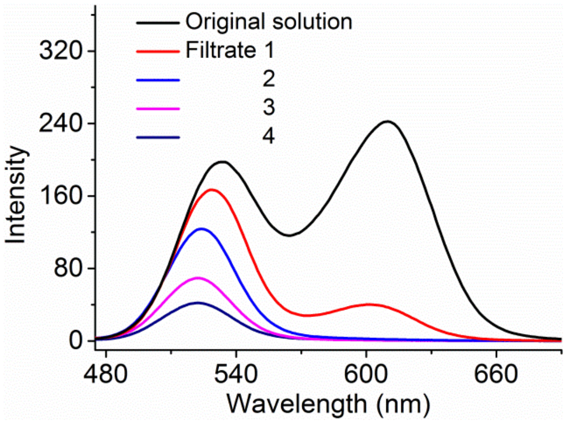

Filtrate entry	Membrane thickness[a] (μm)	Luminescence λ_{max} (nm)	FWHM (nm)	Separation efficiency (%)
1	0.20~0.35	529/602	40.2	–
2	0.43	523	39.0	72.3
3	1.43	523	39.1	40.5
4	2.19	522	39.0	25.2

[a]The thicknesses of Tr-membranes are estimated from Supplementary Fig. 29.

Supplementary Figure 47: Fluorescent spectra and membrane thickness dependence. (Top) The solution of TG-1 (λ_{max} = 533 nm, D = 3.3 nm) and TG-2 (λ_{max} = 611 nm, D = 4.4 nm) QDs mixture and the filtrates screened by Tr-membranes with thickness at: 0.20~0.35 (Filtrate 1), 0.43 (Filtrate 2), 1.43 (Filtrate 3) and 2.19 μm (Filtrate 4). (Down) The filtration analysis on membrane thickness via emission bands (λ_{max}), full width at half maximum (FWHM) and separation efficiency.

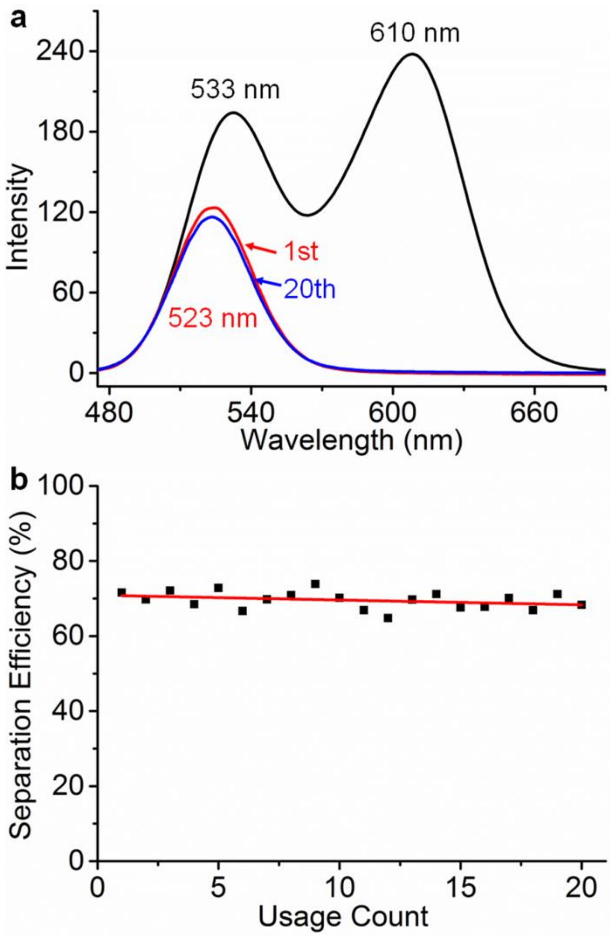

Supplementary Figure 48: Continuous separations. (**a**) Fluorescent spectra of virgin QDs mixture solution of TG-1 (λ_{max} = 533 nm, D = 3.3 nm) and TG-2 (λ_{max} = 611 nm, D = 4.4 nm) (black line), the 1st (red line) and 20th (blue line) filtrates by using the same Tr-membrane; (**b**) the plot of separation efficiency versus 20-mL dosage count of the same Tr-membrane.

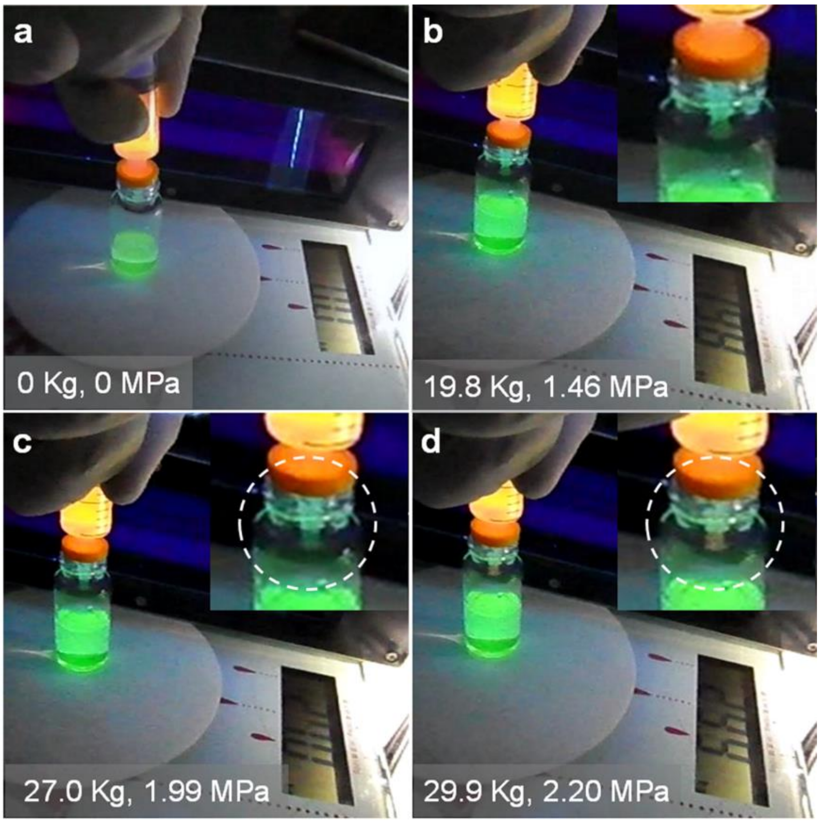

Supplementary Figure 49: Crashing test. Digital photographs of QDs mixture solution of TG-1 (λ_{max} = 533 nm, D = 3.3 nm) and TG-2 (λ_{max} = 611 nm, D = 4.4 nm) passing through Tr-membrane with increased stress on syringe plunger: (**a**) 0, (**b**) 194.0 (19.8 kg $\times$ 9.8 N kg^{-1}), (**c**) 264.6 (27.0 kg $\times$ 9.8 N kg^{-1}) and (**d**) 293.0 N (29.9 kg $\times$ 9.8 N kg^{-1}). In this experiment, [Azo-TrEG@CD][PWV] solution (0.04 mg mL^{-1}, 8.3 mL) was injected into a syringe filter (pore size: 0.22 μm, effective area: $\pi\times(0.65)^2$ = 1.33 cm^2, area of the plunger: $\pi\times(0.75)^2$ = 1.77 cm^2) for a Tr-membrane (0.25 mg cm^{-2}). The whole separation installation was placed on an electronic weigher for stress measurement. When the stress increases to ca. 264.6 N, the separation still works well (spectra shown in Supplementary Fig. 50). When increasing the stress to ca. 293.0 N, the luminescence of the filtrate at the needle tip starts to turn orange, implying the damage of Tr-membrane. Thus, the burst strength of the Tr-membrane is estimated to be ca. 1.49−1.66 MPa (calculated from the stress values divided by area of the syringe plunger). Due to the Tr-membrane spreading on a syringe filter, its actual test area is the pore area of syringe filter: ($\pi\times0.0011^2$) = 3.8×10^{-6} cm^2. Because the burst strength is closely related to membrane thickness and the test area, it is difficult to estimate the comparable membrane stability by using a single value and therefore some related parameters of commercial membranes are listed in Supplementary Table 5 for reference.

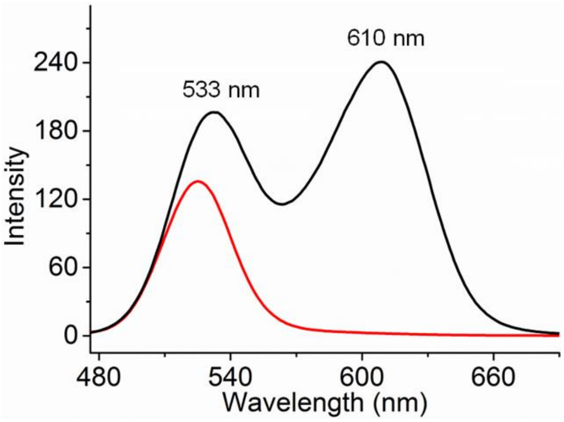

	λ_{max} (nm)	FWHM (nm)	Separation efficiency (%)
Filtrate	524	38.9	75.4

Supplementary Figure 50: Pressure depended fluorescent spectra. Mixture solution of TG-1 and TG-2 QDs (balck line) and the filtrate (red line) screened by Tr-membrane under the pressure of ca. 1.49 MPa and **summary** of emission band (λ_{max}), FWHM and separation efficiency of filtrate.

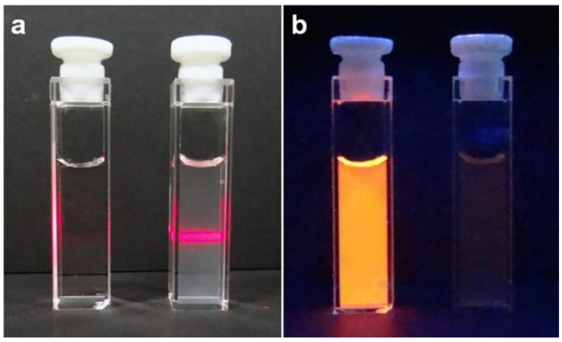

Supplementary Figure 51: Digital photographs. (**a**) Without and (**b**) with 365-nm light irradiation for the blocked QDs washed out from the used Tr-membrane (left) or the dispersion of the used Tr-membrane upon sonication (right). The comparison of photographs shows that most of residual QDs can be taken out from the membrane by simply washing and the used Tr-membrane can be re-dispersed in water.

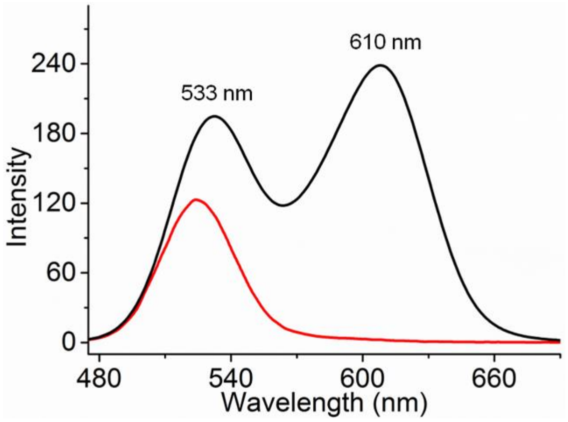

	λ_{max} (nm)	FWHM (nm)	Separation efficiency (%)
Filtrate	524	39.3	69.4

Supplementary Figure 52: Fluorescent spectra. Mixture solution of TG-1 and TG-2 QDs (black line) and its filtrate screened through the Tr-membrane prepared by filtering the re-dispersed solution of used Tr-membrane and **summary** of emission band (λ_{max}), FWHM and separation efficiency of filtrate.

Supplementary Tables

Supplementary Table 1: Summary of assignment for ^{1}H NMR signals. Azo-TrEG ·2Br upon addition of 2.0 eq. CDs in Fig. 2.

Signal	H(a)	H(b)	H(c)	H(d)	H(e)	H(f)	H(g)	H(h)	H(i)	H(j)	H(k)
Chemical Shift (ppm)	0.06	0.09	0.15	0.29	0.23	0.82	0.27	0.39	0.31	0.14	0.07

Supplementary Table 2: Summary of elemental analysis data[a].

		C	H	N	P	W	V
[Azo-TrEG@CD][PWV]	Found (%)	34.06	4.10	2.12	0.36	24.72	0.68
	Calcd. (%)	33.97	4.10	2.09	0.38	25.08	0.63
[Azo-TeEG@CD][PWV]	Found (%)	34.32	4.26	2.04	0.42	24.52	0.64
	Calcd. (%)	34.04	4.19	2.05	0.38	24.70	0.62

[a]The formulae of [Azo-TrEG@CD][PWV] and [Azo-TeEG@CD][PWV] are $\{[(C_{42}H_{42}N_6O_4)(C_{36}H_{60}O_{30})_2]_2[PW_{11}VO_{40}](H_2O)_2\}_n$ and $\{[(C_{44}H_{46}N_6O_5)(C_{36}H_{60}O_{30})_2]_2[PW_{11}VO_{40}](H_2O)_4\}_m$, respectively. Elemental analytical results of C, H and N were obtained from organic elemental analysis, and elemental analysis of P, W and V were performed on ICP-AES.

Supplementary Table 3: Summary of Miller indices and observed diffraction patterns of in-layer structures of [Azo-TrEG@CD][PWV] in Supplementary Fig. 23b.

Sample	Measured 2θ (°)	Measured d (nm)	Fitted Miller indices h	k	l
[Azo-TrEG@CD][PWV]	2.42	3.65	0	0	1
	4.92	1.80	0	0	2
	7.36	1.20	0	0	3
	10.01	0.88	0	0	4
	12.24	0.72	0	0	5
	14.76	0.60	0	0	6
	17.26	0.51	0	0	7
	19.26	0.46	0	0	8
	22.78	0.39	0	0	9

Supplementary Table 4: Summary of the emission band (λ_{max}) and FWHM. Data from the 1st and 20th filtrates treated by using one Tr-membrane.

	λ_{max} (nm)	FWHM (nm)	Separation efficiency (%)
1st	524	39.3	71.6
20th	523	39.2	68.3

Supplementary Table 5: Summary of reported data for reference. Comparison of the prepared IOIF membrane and commercial Whatman membranes.

Materials	Thickness (μm)	Test area[a] (cm^2)	Burst strength (MPa)
IOIF	0.43	3.8×10^{-6}	1.49~1.66
Polycarbonate	7~20	4.9	>0.069
Polyester	9~23	17.3	>0.069
Teflon	130	4.9	0.090
Nylon	150~187	1.3	0.23~0.28
Aluminium oxide	60	1.3	0.45~0.76

[a]Due to the IOIF membrane spreading on a syringe filter, its actual test area is the pore area of syringe filter; while for the commercial membranes, their test areas are the whole area of membranes.

Supplementary References

1. Polarz, S., Smarsly, B. & Antonietti, M. Colloidal organization and clusters: self-assembly of polyoxometalate-surfactant complexes towards three-dimensional organized structures. *ChemPhysChem* **7**, 457–461 (2001).
2. Leroy, F., Miró, P., Poblet, J. M., Bo, C. & Ávalos, J. B. Keggin polyoxoanions in aqueous solution: ion pairing and its effect on dynamic properties by molecular dynamics simulations. *J. Phys. Chem. B* **112**, 8591–8599 (2008).
3. Ito, T., Sawada, K. & Yamase, T. Crystal structure of bis(dimethyldioctadecylammonium) hexamolybdate: a molecular model of langmuir–blodgett films. *Chem. Lett.* **32**, 938–939 (2003).
4. Nyman, M. *et al.* Comparative study of inorganic cluster-surfactant arrays. *Chem. Mater.* **17**, 2885–2895 (2005).
5. Ito, T. & Yamase, T. Inorganic-organic hybrid layered crystal composed of polyoxomolybdate and surfactant with π Electrons. *Chem. Lett.* **38**, 370–371 (2009).
6. Yang, Y., Wang, Y., Li, H., Li, W. & Wu, L. Self-assembly and structural evolvement of polyoxometalate-anchored dendron complexes. *Chem. Eur. J.* **16**, 8062–8071 (2010).
7. Nisar, A., Lu, Y., Zhuang, J. & Wang, X. Polyoxometalate nanocone nanoreactors: magnetic manipulation and enhanced catalytic performance. *Angew. Chem. Int. Ed.* **50**, 3187–3192 (2011).
